# Dual interfacial engineering of a Chevrel phase electrode material for stable hydrogen evolution at 2500 mA cm^−2^

**DOI:** 10.1038/s41467-022-34121-y

**Published:** 2022-10-26

**Authors:** Heming Liu, Ruikuan Xie, Yuting Luo, Zhicheng Cui, Qiangmin Yu, Zhiqiang Gao, Zhiyuan Zhang, Fengning Yang, Xin Kang, Shiyu Ge, Shaohai Li, Xuefeng Gao, Guoliang Chai, Le Liu, Bilu Liu

**Affiliations:** 1 Shenzhen Geim Graphene Center, Tsinghua-Berkeley Shenzhen Institute & Shenzhen International Graduate School, Tsinghua University, Shenzhen, 518055 P. R. China; 2 Institute of Materials Research, Shenzhen International Graduate School, Tsinghua University, Shenzhen, 518055 P. R. China; 3 State Key Laboratory of Structural Chemistry, Fujian Institute of Research on the Structure of Matter, Chinese Academy of Sciences, Fuzhou, 350002 P. R. China; 4 Functional Materials and Interfaces Lab, Suzhou Institute of Nano-Tech and Nano-Bionics, Chinese Academy of Sciences, Suzhou, 215123 P. R. China; 5 School of Nano-Tech and Nano-Bionics, University of Science and Technology of China, Hefei, 230026 P. R. China

**Keywords:** Mechanical and structural properties and devices, Electrocatalysis, Two-dimensional materials

## Abstract

Constructing stable electrodes which function over long timescales at large current density is essential for the industrial realization and implementation of water electrolysis. However, rapid gas bubble detachment at large current density usually results in peeling-off of electrocatalysts and performance degradation, especially for long term operations. Here we construct a mechanically-stable, all-metal, and highly active CuMo_6_S_8_/Cu electrode by in-situ reaction between MoS_2_ and Cu. The Chevrel phase electrode exhibits strong binding at the electrocatalyst-support interface with weak adhesion at electrocatalyst-bubble interface, in addition to fast hydrogen evolution and charge transfer kinetics. These features facilitate the achievement of large current density of 2500 mA cm^−2^ at a small overpotential of 334 mV which operate stably at 2500 mA cm^−2^ for over 100 h. In-situ total internal reflection imaging at micrometer level and mechanical tests disclose the relationships of two interfacial forces and performance of electrocatalysts. This dual interfacial engineering strategy can be extended to construct stable and high-performance electrodes for other gas-involving reactions.

## Abstract (web summary)

Stable electrodes which operate at large current density are essential for industrial water electrolysis. Here, a highly active Chevrel phase electrode is reported to achieve 2500 mA/cm^−2^ current density for 300 hours at small overpotentials.

## Introduction

The extensive use of fossil fuel has caused environmental pollution and energy crisis. Especially, carbon emissions from fossil fuel contribute ~65% of the total global emissions of greenhouse gases^1^ and it is thus urgent to develop green energy. Due to the superiorities of high energy efficiency, high mass-energy density, and zero-carbon emissions^2^, green hydrogen produced by electrochemical water splitting is promising as future clean energy carrier. However, electrocatalysts applied in industrial water splitting have to suffer from harsh conditions, such as large current density, long working period, high pressure and elevated temperature^3–6^, which bring challenges to the mechanical stability of electrodes and making widespread implementation of electrolysis difficult. The mechanical stability of an electrode is mostly determined by two interfaces, i.e., the electrocatalyst-support interface and electrocatalyst-bubble interface. The detachment of many gas bubbles at large current density would produce a strong electrocatalyst-bubble adhesion force^7–10^, which can peel off the electrocatalysts when it is larger than the binding force between the electrocatalyst and support. Therefore, it is important to enhance mechanical stability of electrodes by enhancing electrocatalyst-support and weakening electrocatalyst-bubble interfacial forces.

Along this direction, the most common way to enhance the adhesion of electrocatalysts on supports is using binders such as Nafion, but it usually cannot provide an adhesion force that is large enough to stand bubble bombardment^11^. Moreover, the use of binder may also block active sites, reduce ionic conductivity and make the electrode aerophilic^12–15^, which are harmful to reaction kinetics and bubble detachment. To avoid these side effects, many methods to in situ grow electrocatalysts on supports have been developed to construct binder-free self-supporting electrodes^16–19^. Such in situ growth can well control the morphology of electrode to promote bubble detachment and reduce the electrocatalyst-bubble adhesion force^20–22^. Wet chemical synthesis and vapor phase deposition are two common in situ growth methods. So far, most of the thus grown electrocatalysts adhere to the supports mainly via weak physical or chemical interactions, including electrostatic adsorption, mechanical interlocking of porous structure and intermolecular attraction induced by van der Waals force^23,24^. Such weak interface between electrocatalyst and support is usually difficult to stand bubbling^25,26^, and leads to poor stability of electrode. In addition, in the case of widely-used semiconducting electrocatalysts^27,28^, the contact between semiconducting electrocatalyst and metal support commonly shows Schottky barrier at interface, bringing high contact resistance and retarding reaction kinetics^29–32^. Therefore, developing synthesis method that can produce metallic, highly active electrode with strong electrocatalyst-support interface and weak electrocatalyst-gas interface is important for gas-involving electrocatalysis.

Here, we develop a mechanically stable all-metal CuMo_6_S_8_/Cu electrode, of which Chevrel phase CuMo_6_S_8_ derived from 2H-phase MoS_2_ and in-situ grown on Cu foam. In previous work, nano-Chevrel phase^33^ and layered Cu_2_S/Cu_2.76_Mo_6_S_8_/MoS_2_ heterojunction^34^ were synthesized to study their HER activity. These catalysts delivered geometrical current densities of 20 and 100 mA cm^−2^, which are relatively small. By optimizing the electrode structure, high performance and good stability of Chevrel phase catalysts at large current density could be realized. In this work, our CuMo_6_S_8_/Cu electrode has a strong electrocatalyst-support interfacial binding force, a weak electrocatalyst-bubble adhesion force, and all-metal property, which effectively avoid peeling-off of electrocatalysts and improve reaction kinetics at large current density. As a result, the electrode has superior HER performance with a small overpotential of 334 mV to reach 2500 mA cm^−2^ and operates stably at 2500 mA cm^−2^ for over 100 h. In addition, we use the in-situ total internal reflection imaging method to visualize the peeling-off of electrocatalysts at large current density with a micrometer spatial resolution, showing that the peeling-off degree of electrocatalysts on CuMo_6_S_8_/Cu electrode is four times smaller than that on Pt/C electrode. Such distinguished stability of CuMo_6_S_8_/Cu electrode is because it shows an interfacial binding force twice of Pt/C electrode and an electrocatalyst-bubble adhesion force half of Pt/C electrode. The highly active sites and metallic properties of CuMo_6_S_8_ also guarantee superior HER performance at large current density. The dual interfacial engineering developed here can in principle be extended to construct stable and high-performance electrodes for other gas-involving reactions.

## Results

### Preparation and characterization of the Chevrel phase CuMo_6_S_8_/Cu electrode

**Fig. 1:**
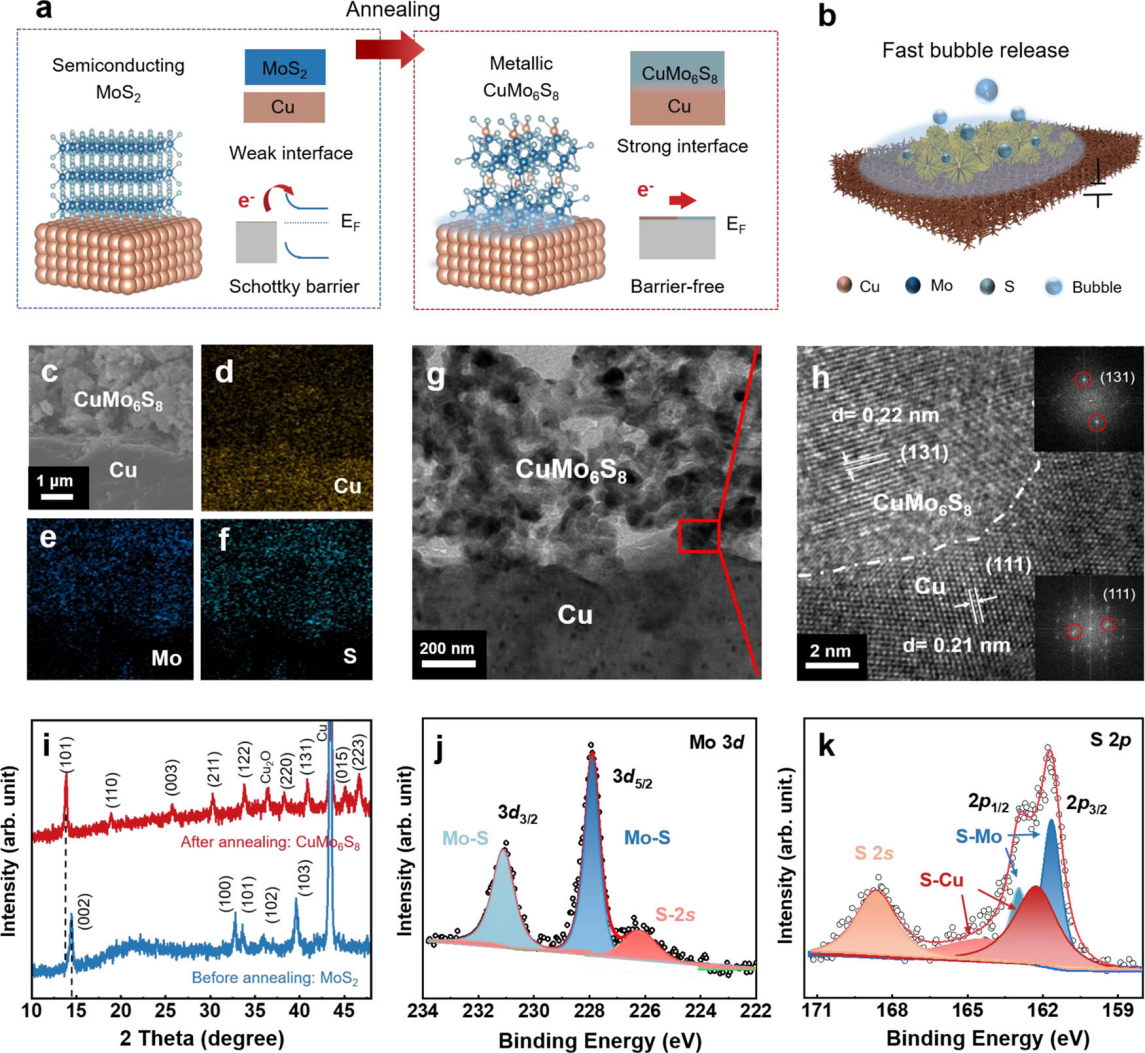
Preparation and characterization of the Chevrel phase CuMo_6_S_8_/Cu electrode. **a**, **b** A schematic showing the preparation process and macro-performance of CuMo_6_S_8_/Cu electrode. **c**–**f** Scanning electron microscopy (SEM) images and energy dispersive X-ray spectroscopy (EDS) mappings of a cross-section of the electrode. **g** Cross-sectional high-resolution transmission electron microscopy (HRTEM) image of the CuMo_6_S_8_/Cu electrode. **h** The zoom-in view taken from the red rectangular area in **g**, showing the interface between the CuMo_6_S_8_ layer and Cu substrate. The insets of **h** are the corresponding fast Fourier transform patterns for CuMo_6_S_8_ (top) and Cu (bottom). **i** X-ray diffraction patterns of the samples before and after annealing. **j**, **k** X-ray photoelectron spectroscopy spectra of Mo 3*d* (**j**) and S 2*p* (**k**) of CuMo_6_S_8_.

The CuMo_6_S_8_/Cu electrode is prepared by intermediate-assisted grinding exfoliation of bulk MoS_2_ to produce 2D MoS_2_^35^, followed by loading them on Cu foam and high-temperature annealing (Fig. 1a, Methods and Supplementary Fig. 1). MoS_2_ nanoflakes with an average thickness of 36 nm are obtained (Supplementary Figs. 2–3), and X-ray diffraction (XRD) and Raman spectroscopy results show that its composition and phase show negligible change after exfoliation (Supplementary Fig. 3). Before annealing, there are only physical contact and weak electrostatic adsorption between the MoS_2_ nanoflakes and Cu foam support. The Schottky barrier will form at the interface because of metal-semiconductor contact, which will impede electron transfer from support to electrocatalyst. To strengthen the interfacial binding and eliminate this Schottky barrier, the electrode is annealed at 750 °C under Ar and H_2_ atmosphere. This process may also lead to the Wenzel-state wetting property and realize fast gas bubble detachment in terms of macro-performance (Fig. 1b). After annealing, scanning electron microscopy (SEM) images show that the morphology of materials changes from agglomerated nanoflakes to porous structure, which composed of nanoflakes or nanoparticles and a few nanowires. (Supplementary Figs. 2d and 4). The morphological diversity of the material may be due to the low melting point and high-temperature diffusion of Cu. Energy dispersive X-ray spectroscopy elemental mappings of a cross-section of the electrode indicate that Cu atoms react with MoS_2_ (Fig. 1c–f) and in-situ form Chevrel phase CuMo_6_S_8_ during annealing, which is also confirmed by high-resolution transmission electron microscopy (HRTEM) image. Figure 1g shows the cross-sectional HRTEM image of the CuMo_6_S_8_/Cu electrode prepared by focused ion beam cutting. The zoom-in view of Fig. 1h shows the interface between CuMo_6_S_8_ layer and Cu substrate. The lattice spacings of 0.22 nm and 0.21 nm correspond to (131) and (111) planes of CuMo_6_S_8_ and Cu, respectively. The insets are the corresponding FFT patterns, which also show the crystalline nature of CuMo_6_S_8_ and Cu. The dotted line indicates the interface between them, which is smooth, indicating a close contact between CuMo_6_S_8_ and Cu with robust interface. Both SEM and TEM characterization demonstrate that there is a well-defined robust interface between CuMo_6_S_8_ and Cu. In addition, HRTEM images of other crystal facets of CuMo_6_S_8_ are provided in Supplementary Fig. 5. The XRD patterns show that after annealing, the 2H-phase MoS_2_ is completely converted into Chevrel phase CuMo_6_S_8_ (PDF #34-1379) with the main diffraction peaks of (101) at 13.7°, (003) at 25.6°, and (131) at 40.8° (Fig. 1i and Supplementary Fig. 7, 8). The peaks from Cu and Cu_2_O are also observed because electrocatalysts are loaded on Cu foam. The zoom-in inspection of the peaks in 2 theta of 10–20° is shown in Supplementary Fig. 6. Besides, Raman peaks including E_g_ (126, 145, 360, 384 cm^−1^) and A_g_ (202, 220, 285 cm^−1^) are found, which correspond to the characteristic peaks of Chevrel phase (Supplementary Fig. 9)^36^. The stoichiometric ratio of Mo to S is 0.73, as measured by the inductively coupled plasma optical emission spectrometer (ICP-OES, Supplementary Table 1), suggesting the formation of pure CuMo_6_S_8_ with a theoretical Mo to S ratio of 0.74. In addition, X-ray photoelectron spectroscopy (XPS) survey spectrum is also implemented to detect the chemical bonding of the CuMo_6_S_8_ layer that cover the surface of Cu foam, and shows that there are five elements (Cu, Mo, S, C and O) in the sample (Supplementary Fig. 10). The Mo 3*d* spectrum shows that two peaks are located at 231.1 eV and 227.9 eV from 3*d*_3/2_ and 3*d*_5/2_, which are originated from Mo-S bonds in CuMo_6_S_8_^37^ (Fig. 1j). The deconvoluted peaks of S 2*p* spectrum can be assigned to S-Mo and S-Cu bonds, which locate at 162.9 eV and 161.6 eV for S-Mo bonds, 164.6 eV and 162.4 eV for S-Cu bonds^38,39^ (Fig. 1k). Taken together, the above results show that the Chevrel phase CuMo_6_S_8_ is synthesized by in-situ reaction of MoS_2_ and Cu foam, which binds tightly to the support by chemical bonding.

### HER performance at large current density

**Fig. 2:**
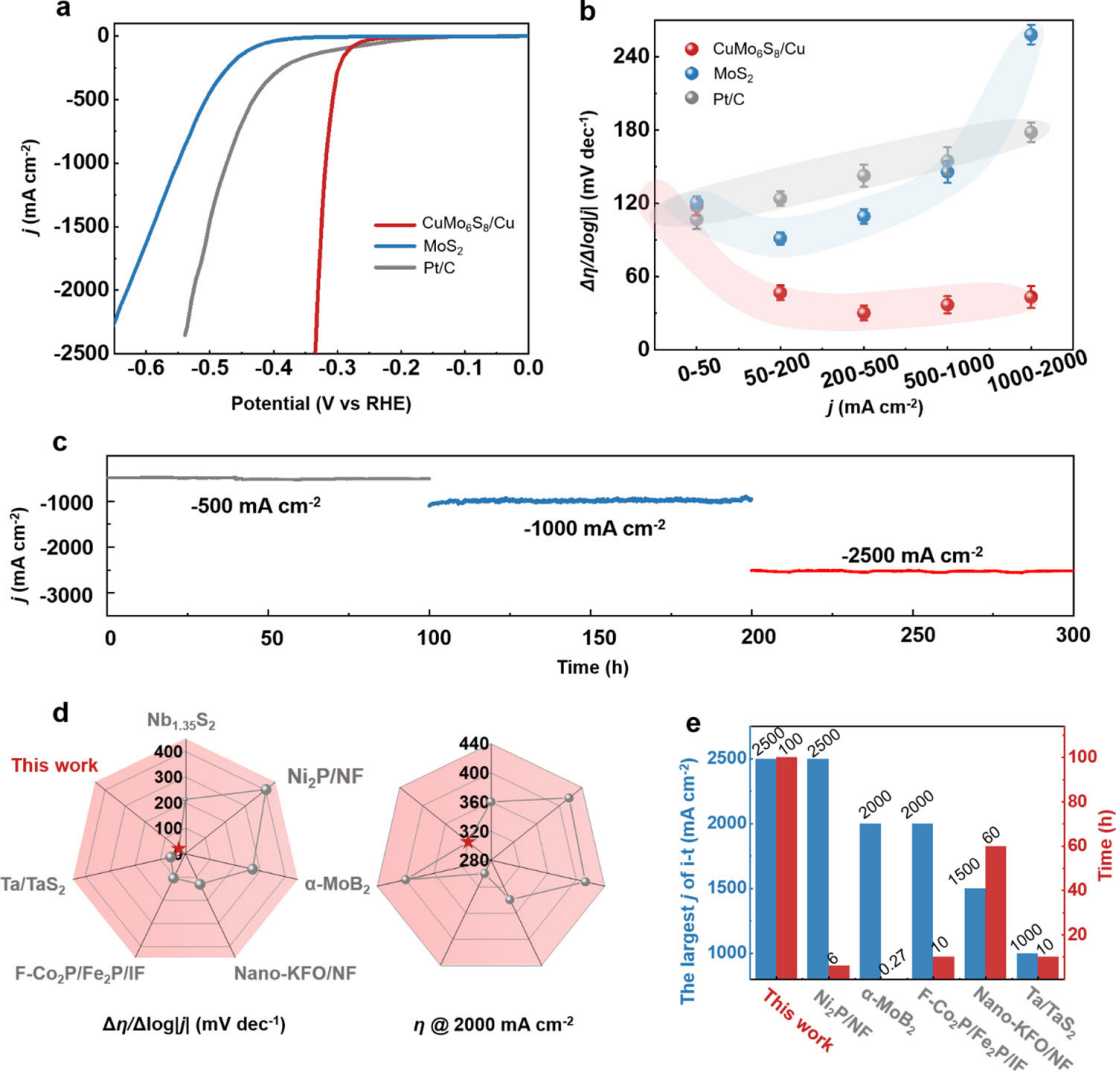
HER performance at large current density. **a** Polarization curves of three electrodes including CuMo_6_S_8_/Cu, MoS_2_, and Pt/C. All tests are done in 1 M KOH at a scan rate of 1 mV s^−1^ with 85% *iR* correction. **b** Δ*η*/Δlog|*j* | ratios of three electrodes at different current density ranges. **c** Chronoamperometric (i-t) curves of the CuMo_6_S_8_/Cu electrode at current densities of −500, −1000, −2500 mA cm^−2^ over 300 h, where the current densities correspond to potentials of −0.43 V, −0.57 V, −0.94 V vs RHE without *iR* correction, respectively. **d**, **e** Comparisons of HER performance and stability of the Cu/CuMo_6_S_8_ electrode with non-noble metal electrocatalysts operated at a current density above 2000 mA cm^−2^. From left to right: the Δ*η*/Δlog|*j* | ratio @1000 mA cm^−2^−2000 mA cm^−2^ and *η @*2000 mA cm^−2^ (**d**) and the largest tested *j* of i-t test and its corresponding time (**e**).

Next, we study the HER performance and stability at large current density in alkaline electrolytes. Figure 2a shows that the overpotentials of CuMo_6_S_8_/Cu electrode are only 320 mV at 1000 mA cm^−2^ and 334 mV at 2500 mA cm^−2^, which are much smaller than that of MoS_2_ (579 mV @1000 mA cm^−2^) and Pt/C (474 mV @1000 mA cm^−2^) electrodes. There is only 14 mV deviation of overpotential from 1000 mA cm^−2^ to 2500 mA cm^−2^, showing a small energy barrier for the CuMo_6_S_8_/Cu electrode operating at large current density. The overpotential of CuMo_6_S_8_/Cu electrode at 10 mA cm^−2^ is 172 mV, and the intrinsic activity of CuMo_6_S_8_ is obtained by normalizing electrochemical surface area (ECSA) and mass of electrocatalyst (Supplementary Figs. 11c–f). The performance of Pt/C electrode is also provided as a reference. Here the Pt/C electrode is prepared by dropping Pt/C ink on Cu foam, which shows similar performance compared with literature (Supplementary Table 2). To gain insights into performance at large current density, the slope of the polarization curve Δ*η*/Δlog|*j*|is implemented to evaluate the performance of the electrode at large current density (Fig. 2b)^40^. The Δ*η*/Δlog*|j* | ratios of the MoS_2_ and Pt/C electrodes increase sharply with increasing current density, while that of the CuMo_6_S_8_/Cu electrode maintains a small value of 43 mV dec^−1^, indicating its better mass transfer ability at large current density. In addition, the stability of the CuMo_6_S_8_/Cu electrode is tested by chronoamperometric (i-t) method at large current densities of 500, 1000, 2500 mA cm^−2^ (Fig. 2c). The i-t curves show negligible degradation of performance within 300 h, especially the stability can be maintained at an ultra-large current density of 2500 mA cm^−2^ for over 100 h, suggesting this electrode has an extremely stable structure.

For hydrogen evolution at large current density, interfacial charge transfer between catalyst and support significantly affects catalytic kinetics. Electrochemical impedance spectra show that the charge transfer resistance of CuMo_6_S_8_/Cu is twice and three times less than that of the MoS_2_ and Pt/C electrodes. (Supplementary Fig. 11a). This is because the interfacial Schottky barrier has been eliminated in CuMo_6_S_8_/Cu due to the transformation from semiconductor (MoS_2_) to metal (CuMo_6_S_8_) (Fig. 5f). Besides, charge transfer resistance can be reduced further because we do not use Nafion binder compared with Pt/C electrode. Then, we compare with the reported state-of-art non-noble metal electrocatalysts operated at current density above 2000 mA cm^−2^ from three aspects, that the Δ*η*/Δlog|*j*|ratio @ 1000 mA cm^−2^–2000 mA cm^−2^, *η* @ 2000 mA cm^−2^ and the largest tested *j* of i-t test and its corresponding operation time (Fig. 2d, e, and Supplementary Table 3). It is seen that the Δ*η*/Δlog|*j*| ratio of CuMo_6_S_8_/Cu electrode is the smallest (32.5 mV dec^−1^) and the *η* @ 2000 mA cm^−2^ of our work (324 mV) approaching the best one reported. Moreover, the tested *j* of i-t test (2500 mA cm^−2^) and its corresponding operation time (100 h) of our work are the largest and longest compared with reported literature. Besides, our work is also better than most other reported electrocatalysts operating at a current density of 500–1500 mA cm^−2^ in terms of performance and stability. (Supplementary Fig. 12, Table 4). The above results show superior electrochemical performance and excellent stability of the CuMo_6_S_8_/Cu electrode at large current density, which is attributed to the unimpeded interfacial charge transfer, abundant active sites and strong interfacial binding.

### In-situ total internal reflection imaging method to characterize mechanical stability of electrode

**Fig. 3:**
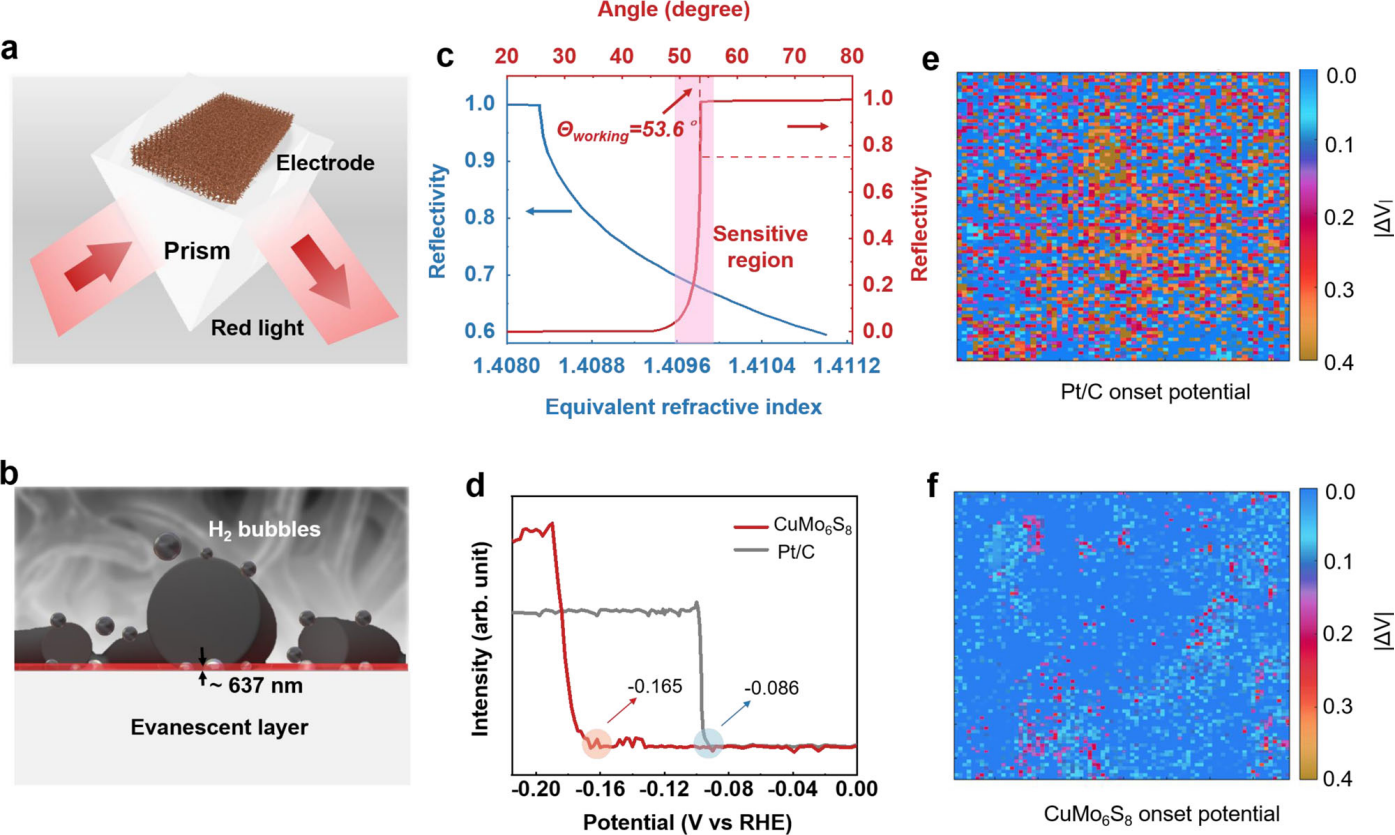
In-situ total internal reflection imaging method to characterize electrode stability. **a** Schematic of the macroscopic contact among the red light, prism and electrode. The incident red light is at the TIR angle. **b** The new-born microscopic interfaces between the electrode, H_2_ bubbles, and electrolyte during HER. The evanescent layer is about 637 nm. **c** The relationships between incident angle, equivalent refractive index and reflectivity. **d** The relationships between onset potential of electrode and light intensity. **e**, **f** Difference value mappings of the onset potential of Pt/C and CuMo_6_S_8_/Cu electrode before and after 10,000 CV cycles.

Then, we use an optical method of in-situ total internal reflection (TIR) imaging method^41,42^ to get insightful information about the variation of the electrode activity. This electrode experiences 10,000 cyclic voltammetry (CV) cycles from 0 to −1500 mA/cm^2^ to quantify the electrode stability at large current density. The schematic of our home-made TIR sensor system, its explosive views, and photos are shown in Supplementary Fig. 13. This system mainly consists of incident light module, detection module, and electrochemical cell module (see Methods for more details). The electrode is in close contact to a prism and the red light is incident at the interface between the prism and electrolyte at a critical TIR angle (Fig. 3a). Immediately when hydrogen evolution occurs, new microscopic interfaces will form between the electrode, H_2_ bubbles and electrolyte in the evanescent layer (Fig. 3b). This working angle (*Θ*_working_) is set a little smaller than the critical TIR angle, which is 53.6° corresponding to the reflectivity of 0.75 in its sensitive region (Fig. 3c). The generated H_2_ bubbles make the equivalent refractive index decrease, accompanied by a decrease in the intensity of the reflected light (Fig. 3c, Eq. (1)). The equivalent refractive index can be approximately expressed as:

1 $${n}_{{{{{{\rm{equivalent}}}}}}}=\left(1-v\right)\times {n}_{{{{{{\rm{electrolyte}}}}}}}+v\times {n}_{{{{{{\rm{bubble}}}}}}}$$

Where *n*_equivalent_ is the equivalent refractive index, *n*_bubble_ and *n*_electrolyte_ is the refractive index of H_2_ bubble (1.000) and KOH electrolyte (1.409), respectively, *v* is the volume concentration of H_2_ bubbles in the evanescent layer. As shown in Fig. 3c, few bubbles generated (*v* = 0.17%) in the evanescent layer can cause a large reduction of reflectivity. Therefore, the potential corresponding to mutation of reflective light intensity in one pixel can be defined as onset potential. It is worth noting that in this method, the onset potential results are obtained in the early instantaneous process, where the microbubbles are invisible due to their small sizes and very small amounts. This is like a “turn on” moment of the HER reaction. Note that the growth of microbubbles into big bubbles, as well as the subsequent diffusion and adhesion of bubbles to the prism all occur after the process of obtaining onset potential data, so it would not affect the test results (Supplementary Movie 1). As shown in Fig. 3d, the onset potentials of the Pt/C and CuMo_6_S_8_/Cu electrodes are −86 and −165 mV vs. reversible hydrogen electrode (RHE), which agrees well with the electrochemical test results and confirms the reliability of TIR imaging method. Here, the onset potential information of the pixels in the whole electrode area before and after 10,000 CV cycles is in-situ collected and combined into mappings. The pristine views and onset potential mappings before and after 10,000 CV cycles are shown in Supplementary Figs. 14, 15. Mappings of difference values of onset potentials of the Pt/C and CuMo_6_S_8_/Cu electrodes are shown in Fig. 3e, f. Such difference values stem from electrocatalyst peel-off, and large difference values indicate severe peel-off. We find that the area of large difference values over 0.05 V occupies about 75% field of the Pt/C electrode. Inversely, the area of small difference values below 0.05 V accounts for about 82% for the CuMo_6_S_8_/Cu electrode (Supplementary Fig. 16b). This result show that the mechanical stability of the CuMo_6_S_8_/Cu electrode is much better than that of Pt/C and the interfacial binding through chemical covalent bonding is stronger than the commercial binder. In addition, the results above and the repeatability of the experiments are also verified by three parallel experiments for different CuMo_6_S_8_/Cu and Pt/C electrodes (Supplementary Figs. 17–19), as well as three replicate experiments on the same CuMo_6_S_8_/Cu electrode (Supplementary Fig. 20). Such stability of the CuMo_6_S_8_/Cu electrode is also confirmed by the XRD, polarization curves and ICP-OES of dissolved matters in the solution after i-t test, none of which shows noticeable change (Supplementary Fig. 21, Table 5, 6). Compared with traditional electrochemical methods, the TIR method not only gives the spatial information of activity distribution of the electrode at micrometer level, but also quantifies the peeling-off degree of electrocatalysts well, strongly confirming the mechanical stability of the CuMo_6_S_8_/Cu electrode. In addition, the TIR method also has unique advantages of large field of view, high detection sensitivity for hydrogen bubble formation (Supplementary Table 7, Note 3), easy operation and low requirements for equipment, compared with other optical imaging methods such as X-ray imaging.

### Strong interfacial binding force and weak electrocatalyst-gas bubble adhesion force

**Fig. 4:**
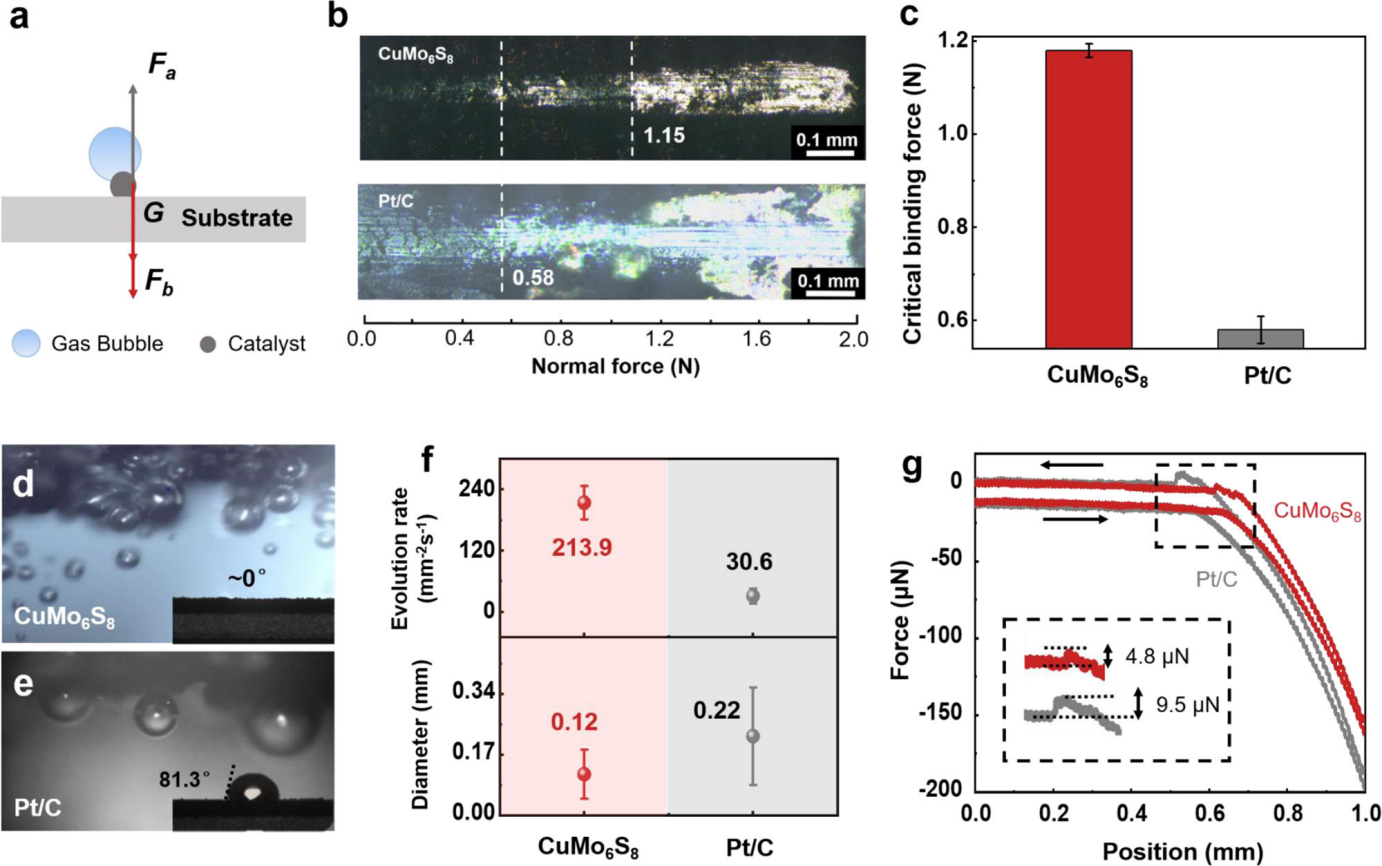
Strong interfacial binding force and small electrocatalyst-gas bubble interfacial adhesion force. **a** Force analysis model of gas bubbles attached on the electrode. *F*_a_*, F*_b_, and *G* represent the electrocatalyst-bubble interfacial adhesion force, the electrocatalyst-support interfacial binding force, and the gravity of electrocatalysts, respectively. **b** The photos of micro-scratches of CuMo_6_S_8_ (top), Pt/C (bottom) catalyst layers adhesive on Cu foils. The dotted lines represent the critical adhesive forces corresponding catalysts. **c** The statistical data of critical adhesive forces of CuMo_6_S_8_ and Pt/C. The error bars represent the statistical distribution of three samples. The photos of gas bubbles generated on **d** CuMo_6_S_8_ and **e** Pt/C electrodes at 10 mA cm^−2^. The insets show the corresponding contact angles of ~0° and 81.3°. **f** Evolution rate and diameter of gas bubbles of CuMo_6_S_8_ and Pt/C electrodes. The error bars represent the distribution of statistical values. **g** Electrocatalyst-bubble interfacial adhesion force of CuMo_6_S_8_/Cu and Pt/C electrodes.

Here, we explain the reasons for excellent performance and mechanical stability of the CuMo_6_S_8_/Cu electrode from two aspects of electrocatalyst-support interfacial binding and electrocatalyst-gas bubble interfacial adhesion force. A simplified force analysis model of gas bubbles attached on the electrode is shown in Fig. 4a. The three forces, including the electrocatalyst-bubble interfacial adhesion force *F*_a_, the electrocatalyst-support interfacial binding force *F*_b_, and the gravity of electrocatalysts *G* are in equilibrium at rest. The electrocatalysts can be adhesive on the support steadily by increasing *F*_b_ and decreasing *F*_a_. First we study the critical binding forces of the CuMo_6_S_8_ and Pt/C electrodes by micro scratch tester. The needle passes across the surface of the coating with an increasing normal force and the coating will begin to peel off at a certain normal force, which is defined as critical binding force. The tested results are confirmed by optical microscopy, friction-normal force curves and acoustic signals^43^ (Fig. 4b, c and Supplementary Fig. 22). The CuMo_6_S_8_ peels off from the support at a load of 1.15 N while 0.58 N for Pt/C. The statistical data from multiple experiments confirm that the CuMo_6_S_8_/Cu electrode has stronger interfacial binding force than the Pt/C electrode.

Second, we in situ observe gas bubble evolution at 10 mA cm^−2^ in a home-made cell by optical microscopy. We find that smaller gas bubbles release quickly from the CuMo_6_S_8_/Cu electrode, while bubbles tend to agglomerate into bigger ones on the Pt/C electrode (Fig. 4d, e and Supplementary Movie 2). The insets of Fig. 4d, e show the contact angles of CuMo_6_S_8_ (~0°) and Pt/C (81.3°) electrodes, manifesting the CuMo_6_S_8_/Cu electrode is more hydrophilic than the Pt/C electrode. Figure 4f shows evolution rate of gas bubbles on the CuMo_6_S_8_/Cu electrode is 213.9 mm^−2^ s^−1^, much higher than that on the Pt/C (30.6 mm^−2^ s^−1^), but the bubble diameter is smaller (0.12 mm *vs*. 0.22 mm). Here we can combine theoretical derivation and experimental data to quantitatively analyze the *F*_a_. Whether the bubbles can be attached to the electrocatalysts depends on the change of the interfacial energy before and after attachment, defined as ΔW, as expressed by Eq. (2):

2 $$\Delta {{{{{\rm{W}}}}}}={\sigma }_{{GL}}\left({\cos }\theta -1\right),$$

where *σ*_GL_ is the interfacial energy between gas and liquid phases, *θ* is the equilibrium contact angle. Detailed derivations are shown in Supplementary Note 1. It is known from Eq. (2) when the *θ* is near zero degrees, that the electrocatalysts are superhydrophilic, gas bubbles are not inclined to attach to the electrode, which means the *F*_a_ is not large enough to cause the electrocatalysts to peeling-off. This analysis has been confirmed by our electrocatalyst-bubble interfacial adhesion force test (Fig. 4g), which shows the *F*_a_ of the CuMo_6_S_8_/Cu electrode (4.8 μN) is much smaller than that of the Pt/C electrode (9.5 μN). Moreover, HER performance at large current density is largely affected by gas bubble detachment. The growth and accumulation of gas bubbles on the catalyst surface would produce micro-convection, impede ion transport and block active site, causing extra energy consumption namely transport overpotential (*η*_trans_)^44,45^. The results of *η*_trans_ at different current densities are plotted in Supplementary Fig. 23 and detailed derivations are shown in Supplementary Note 2. Obviously, the *η*_trans_ and its increasing trend of the CuMo_6_S_8_/Cu electrode at large current density is much smaller than that of the Pt/C. This is because the CuMo_6_S_8_/Cu electrode has a small catalyst-bubble interfacial adhesion force, thus exhibits faster bubble evolution kinetics. The above results jointly illustrates strong interfacial binding force and small electrocatalyst-bubble interfacial adhesion force are responsible for mechanical stability and performance of the CuMo_6_S_8_/Cu electrode at large current density.

### Active sites and metallic property of CuMo_6_S_8_

**Fig. 5:**
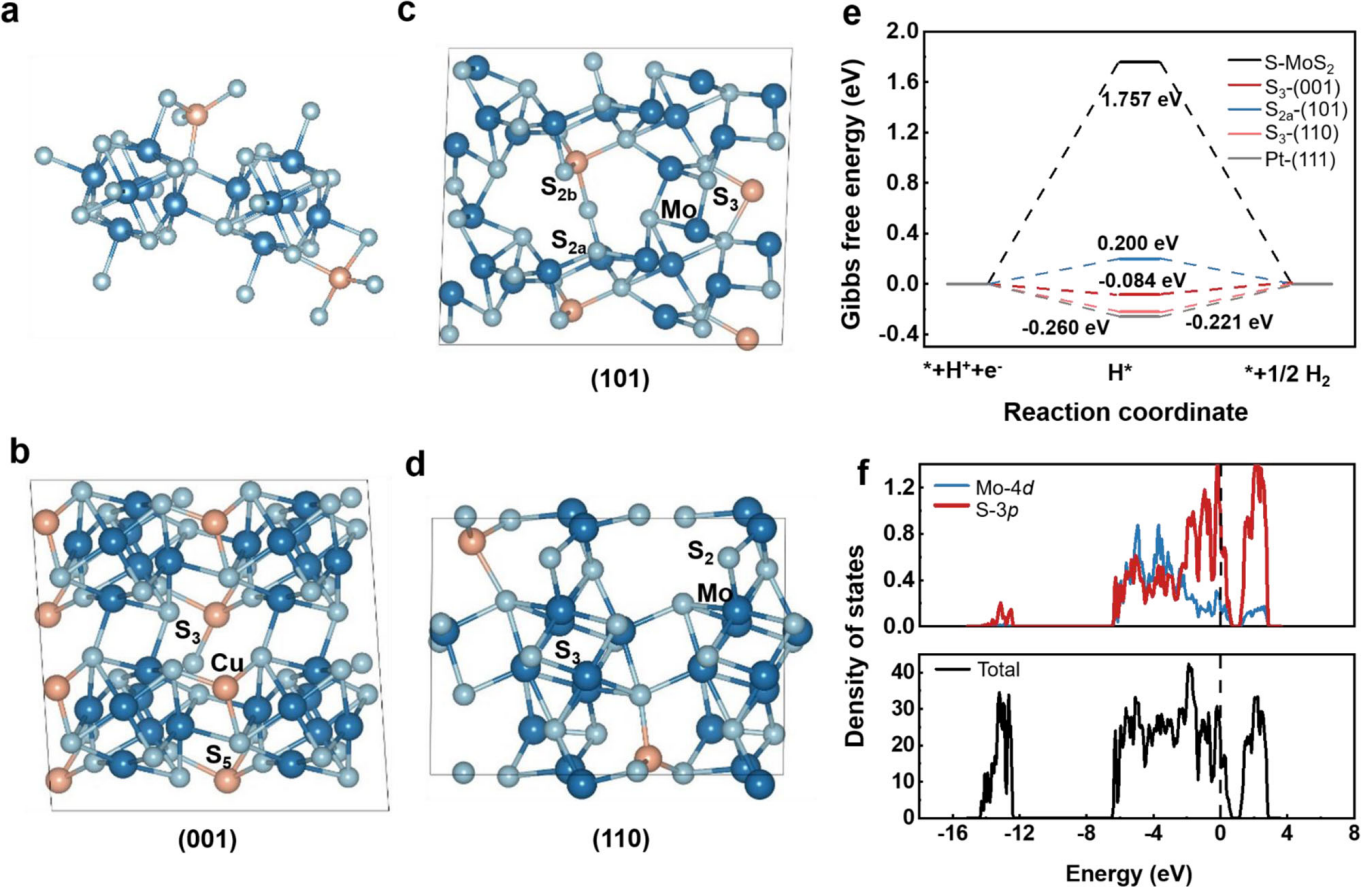
Active sites and metallic property of CuMo_6_S_8_. **a** Atomic structure of CuMo_6_S_8_. **b**–**d** Exposed (001), (101), (110) facets. Orange, dark blue and cyan spheres represent the copper, molybdenum and sulfur atoms, respectively. The marks show the main active sites in different crystal facets and corresponding subscripts represent the coordination number. **e** Gibbs free energy (ΔG_H*_) variations for HER on best active sites of three facets of CuMo_6_S_8_, sulfur site of MoS_2_ and Pt (111). **f** Density of states (DOS) and partial density of states (PDOS) of CuMo_6_S_8_.

The effect of chalcogen electronegativity of Chevrel phase chalcogenides (Mo_6_X_8_; X= S, Se, Te) on HER activity has been investigated previously^46^. Here we further study the relationship between the coordination of sulfur atoms on main crystal facets of CuMo_6_S_8_ and HER activity. Figure 5a–e show the HER active sites on three main facets of CuMo_6_S_8_ and corresponding free energy diagrams. The adsorption free energy of H^*^ (ΔG_H*_) is a reasonable descriptor of HER activity. The ideal HER electrocatalyst should have a moderate ΔG_H*_ which is close to 0 eV. Our calculation results show that pristine MoS_2_ has a bad HER performance with a very high ΔG_H*_, which is consistent with experimental results. S atoms with coordinate numbers of 3, 2 and 3 in CuMo_6_S_8_ (001), (101) and (110) facets have the ΔG_H*_ of −0.084 eV, 0.200 eV and −0.221 eV, respectively (Fig. 5e). All of them are excellent active sites for HER, approaching the ΔG_H*_ of Pt. ΔG_H*_ of other exposed atoms in these facets are also tested, as shown in Supplementary Fig. 24. It is seen that S atoms with lower coordinate numbers usually show lower ΔG_H*_ than those with higher coordinate numbers in each facet. To reveal the causal relationship between the coordinate number of S atom and ΔG_H*_, the Bader effective charges of S atoms with different coordinate numbers are calculated and shown in Supplementary Table 8. The S atom with larger coordinate number shows more negative Bader effective charge. Since the S atom is more electronegative than H in S-H bond, a larger coordinate number of S (already has more negative Bader effective charge) should show a weaker S-H bond strength, i.e., a higher ΔG_H*_. From this perspective, the coordinate number of S atom is a descriptor of HER activity for each facet considered in this study. Noted that this rule only works for S atoms on the same facet but not on different facets. There are plenty of exposed S atoms with coordinate numbers of 2 or 3, which may lead to the excellent HER performance of CuMo_6_S_8_.We also investigate the conductivity of CuMo_6_S_8_ and the density of states (DOS) are shown in Fig. 5f. Total DOS clearly shows that CuMo_6_S_8_ is a metal as the valence bands are partially filled. Partial density of states (PDOS) indicates that the DOS on Fermi energy level mostly originate from Mo-4*d* and S-3*p* states. In comparison, 2H-phase MoS_2_ is a semiconductor as the valence bands are completely filled with electrons and separated from the conduction bands with a band gap (Supplementary Fig. 25). Therefore, a higher electron transfer efficiency in metallic CuMo_6_S_8_ than that on the semiconductor of 2H-phase MoS_2_ is expected. Taken together, the above results suggest that CuMo_6_S_8_ is a metal with high intrinsic HER activity.

## Discussion

In summary, we construct a Chevrel phase CuMo_6_S_8_/Cu electrode with superior mechanical stability and HER performance by dual interfacial engineering. The Chevrel phase CuMo_6_S_8_ originates from MoS_2_ and binds with support firmly by chemical bonding. It features a strong electrocatalyst-support binding force and a weak electrocatalyst-bubble adhesion force. As a result, the CuMo_6_S_8_/Cu electrode achieves excellent performance with a small overpotential of 334 mV@ 2500 mA cm^−2^ and operates at 2500 mA cm^−2^ stably over 100 h. The mechanical stability of the CuMo_6_S_8_/Cu electrode at large current density is quantitatively described by in situ TIR imaging method with a spatial resolution of micrometer, showing peeling-off degree of electrocatalysts on CuMo_6_S_8_/Cu electrode is nearly four times smaller than that of the Pt/C electrode. Furthermore, mechanical tests clarify the influences of interfacial forces on mechanical stability and HER performance at large current density. Theoretical calculations show that CuMo_6_S_8_ has metallic property and high activity, effectively promoting the interfacial electron transport and hydrogen evolution kinetics. The dual interfacial engineering deepens the understanding about the effect of interfacial property on electrode stability and electrochemical performance, which is expected to be used in other gas-involving electrochemical reactions toward constructing stable and highly efficient electrodes.

## Methods

### Material preparations

All chemicals were used as received without further purification. First, we used the intermediate-assisted grinding exfoliation to produce 2D MoS_2_, where bulk MoS_2_ (Aladdin Chemical Reagent Co., 99.5%, diameter <2 μm, 10 g) and Fe powders (serve as force intermediates, Aladdin Chemical Reagent Co., 98%, 400 mesh, 0.01 g) were added together into a mortar grinder (Retsch RM 200, Germany) and ground for 9 h. The sample was further centrifuged with 10,000 rpm, followed by pickling with 1 M HCl and washing with deionized water. Then, the obtained 2D MoS_2_ were dispersed in ethanol and treated by ultrasonic for 10 min. The Cu foams were first treated by CV cycles in 0.5 M H_2_SO_4_ to improve the surface roughness. After that, the MoS_2_ ink was loaded on the treated Cu foams by drop casting or spray coating and the loading amount was 10 mg cm^−2^. Finally, the dried MoS_2_/Cu electrodes were annealed under the Ar (200 sccm) and H_2_ (10 sccm) at 750 °C for 2 h to prepare the CuMo_6_S_8_/Cu electrodes. The Pt/C electrode was obtained by dropping the commercial Pt/C ink (20 wt%) on the Cu foams. The Pt/C ink consisted of 2 mg Pt/C powder (Macklin Biochemical Co., 20%), 0.4 mL isopropanol, 1.5 mL deionized water and 0.1 mL Nafion binder (Dupont Co., 5 wt%). The loading amount of Pt/C was 0.5 mg cm^−2^. The specification of pure Pt foil electrode was 1 × 1 cm^2^.

### Material characterizations

Phase and crystal structure of samples were characterized by XRD (Cu K_α_ radiation, *λ* = 1.54 Å, Bruker D8 Advance, Germany). The surface morphology and elementary composition were characterized by SEM (Hitachi SU8010, 20 kV). HRTEM images were obtained by using an electron acceleration voltage of 300 kV (FEI Tecnai F30, USA). The thicknesses of 2D MoS_2_ nanoflakes were measured by AFM (Oxford Asylum Research, UK). Raman spectra were collected by using a 532 nm laser as excitation light (Horiba LabRAM HR Evolution, Japan). Chemical analysis was performed by high-resolution XPS (monochromatic Al Kα X-rays, Thermo Fisher ESCALAB 250Xi, USA). The mechanical property of the electrodes was characterized by micro scratch tester (Anton Paar, UNHT, Austria). The contact angle was tested by contact angle meter (KRUSS DSA30, Germany). The electrocatalyst-bubble adhesion force was tested by high-sensitivity micro-electrochemical balance (DataPhysics DCAT21, Germany).

### Electrochemical measurements

All electrochemical measurements were conducted by using an electrochemical workstation (Biologic Co., VMP300, France). In all tests, a solution of 1 M KOH electrolytes was used. The Hg/HgO electrode and graphite rod were used as reference and counter electrodes in a standard three-electrode cell. The working and reference electrodes were fixed as close as possible to reduce the solution resistance to be below 0.3 Ω and the wetted area of all tested electrodes was all tailored to be 1 cm^2^ for fair comparison among samples. The following equation was used to convert the applied potential from *vs* Hg/HgO to *vs* RHE:

3 $${{{{{{\rm{E}}}}}}}_{{vs}\,{{{{{\rm{RHE}}}}}}}={{{{{{\rm{E}}}}}}}_{{vs}\,{{{{{\rm{Hg}}}}}}/{{{{{\rm{HgO}}}}}}}+0.059 * {{{{{\rm{pH}}}}}}+0.098 $$

Before tests, the electrolyte was purged with Ar gas for 10 min to exclude oxygen. The linear sweep voltammetry (LSV) was performed at a scan rate of 1 mV s^−1^ with 85% *iR* correction. The cyclic voltammetry (CV) was applied at a scan rate of 50 mV s^−1^. The chronoamperometric (i-t) was applied at a current density of 500, 1000, and 2500 mA cm^−2^ for the 300 h stability tests. Electrochemical impedance spectroscopy was performed at the potential corresponding to a current density of 10 mA cm^−2^ with frequencies from 1 MHz to 0.1 Hz. Electrochemical active surface areas (ECSA) of samples were obtained by dividing the electric double layer capacitance by the specific capacitance. Since the specific capacitances of MoS_2_-based materials are between 20–60 μF cm^−2^ ^1–4^, so we chose 40 μF cm^−2^ to calculate the ECSA. Besides, most Pt-based materials reported showed a specific capacitance between 28 and 60 μF cm^−2^ in alkaline electrolyte^5–8^, so we chose 40 μF cm^−2^ as our specific capacitance for Pt/C and Pt foil.

### TIR measurements

The TIR sensor system (Supplementary Fig. 13a) consisted of three parts, including incident light module, detection module, and electrochemical cell module. In the incident light module, the light emitted by a LED (LR W5AP, Osram, Germany, center wavelength 633 nm, electric power 5 W) was collected by a ×25 objective lens (GCO-2104, Daheng Optics, China) and focused on a home-made 0.3 mm pinhole. The light from the pinhole was collimated by an achromatic convex lens (GCL-010650, Daheng Optics, China, focal length 30 mm), passed a bandpass filter (FL632.8-10, Thorlabs, USA, center wavelength 632.8 nm, bandwidth 10 nm) and a linear polarizer (GCL-050003, Daheng Optics, China, extinction ratio 500:1), then radiated the electrochemical cell module. The light reflected by the electrochemical cell module was collected and digitized by the detection module. The detection module was composed of an imaging lens (HF-5MPB50, YVISION, China, focal length 50 mm, with adapter rings) and a CCD camera (Retiga R3, Qimaging, Canada, 1920 × 1460 pixels, 4.54 × 4.54 µm^2^ pixel size, thermoelectric cooling to −20 °C). The explosive view of the electrochemical cell module was shown in Supplementary Fig. 13b. A Ti wire was inserted from the side of a Cu foam as the working electrode, another Ti wire and an Hg/HgO electrode were the counter electrode and the reference electrode. The photos of the entire TIR system and the electrochemical cell module were shown in Supplementary Fig. 13c–e.

LSV and CV test were performed on the electrochemical module. Before measurements, 1.0 M KOH solution with a volume of 10 mL was injected into the electrolytic cell, and the working electrode was soaked in it followed by vacuuming. Then, the electrochemical cell module was installed in the TIR system and modulated to achieve a working angle of 53.6°. After that, high-resolution images were achieved by adjusting the imaging lens of the detection module. Before 10,000 CV, the first LSV test was initially performed with a scan rate of 1 mV s^−1^ at the potential window of 0 to −0.695 V vs RHE without *iR* correction. Meanwhile, TIR test was also performed with an exposure time of 6 ms and a collection interval of 2 s. Therefore, the electrochemical information and images can be acquired simultaneously to obtain the initial onset potential mapping. After that, the CV test of 10,000 cycles (0 to −1500 mA cm^−2^, w/o *iR* correction, Supplementary Fig. 16a) was performed with a scan rate of 50 mV s^−1^ followed by second vacuuming to exclude accumulated bubbles, and TIR test to acquire the onset potential mapping after 10,000 CV. Post-processing and statistics were performed to compare the numerical difference before and after 10,000 CV.

### Computational methods

All the density functional theory (DFT) calculations were performed via the Vienna Ab initio Simulation package^10–14^, and the projector-augmented plane wave pseudopotentials were used for the elements involved^12^. The generalized gradient approximation of Perdew, Burke, and Ernzerhof was used to treat the exchange correlation between electrons^15^. CuMo_6_S_8_ (110), (001) and (101) surfaces were investigated for HER and the bottom layers kept fixed during the calculation. A vacuum region of greater than 15 Å was added along the direction normal to the slab plane to avoid the interaction between periodic supercells. The electron wave function was expanded in plane waves and a cutoff energy of 500 eV was chosen. The Monkhorst-Pack meshes of (3 × 3 × 1) were adopted for the Brillouin zone of the slabs and primitive cell^16^. The convergence in the energy and force were set to be 10^−4^ eV and 0.01 eV/Å, respectively.

The free energies of H_2_O (l) and H_2_ (g) were used as references when calculating the free energies of reaction intermediates. The adsorption energy for reaction intermediate was calculated as follows^17^:

4 $$\Delta {{{{{\rm{G}}}}}}={\Delta {{{{{\rm{E}}}}}}}_{{{{{{\rm{Total}}}}}}}+{\Delta {{{{{\rm{E}}}}}}}_{{{{{{\rm{ZEP}}}}}}}-{{{{{\rm{T}}}}}}\Delta {{{{{\rm{S}}}}}}+{\Delta {{{{{\rm{G}}}}}}}_{{{{{{\rm{s}}}}}}}$$

where ΔE_Total_ is the calculated adsorption total energy by DFT, ΔE_ZPE_ is zero-point energy, ΔS is entropy, and ΔGs is solvation energy^18,19^. The calculated HER electrochemical potential can be obtained as follows:

5 $${{{{{{\rm{U}}}}}}}_{{{{{{\rm{L}}}}}}}={{{{{\rm{Mini}}}}}}\left[-\Delta {{{{{\rm{Gi}}}}}}\right]/{ne}$$

where *n* is the number of electrons transferred for each electrochemical step, and *e* is the elementary charge. Here, the *n* is set to 1 for the one-electron transfer step. The meaning of the r.h.s. of the above equation is to select the smallest [−ΔG_i_] among the HER elementary steps.

## Supplementary information

Supplementary Information

Peer Review File

Description of Additional Supplementary Files

Supplementary Movie 1

Supplementary Movie 2

## Data Availability

All data are available from the authors upon reasonable request. Source data are provided with this paper.

## Source data

Source Data
