# Supplementary material for: Dual interfacial engineering of a Chevrel phase electrode material for stable hydrogen evolution at 2500 mA cm−2
Source: Nat Commun. 2022 Oct 26;13:6382. doi: 10.1038/s41467-022-34121-y (PMC9605970; doi:10.1038/s41467-022-34121-y)
Supplement: Supplementary file 1 — Supplementary Information [file 41467_2022_34121_MOESM1_ESM.pdf]

Dual interfacial engineering of a Chevrel phase electrode material for stable hydrogen evolution at 2500 mA cm^{-2}

Heming Liu[1, 2, *], Ruikuan Xie[3, *], Yuting Luo[1,2], Zhicheng Cui[2], Qiangmin Yu[1,2], Zhiqiang Gao[4,5], Zhiyuan Zhang[1,2], Fengning Yang[1,2], Xin Kang[1,2], Shiyu Ge[1,2], Shaohai Li[1,2], Xuefeng Gao[4,5], Guoliang Chai[3], Le Liu[2], and Bilu Liu[1, 2*]

1. Shenzhen Geim Graphene Center, Tsinghua-Berkeley Shenzhen Institute & Shenzhen International Graduate School, Tsinghua University, Shenzhen 518055, P. R. China
2. Institute of Materials Research, Shenzhen International Graduate School, Tsinghua University, Shenzhen 518055, P. R. China
3. State Key Laboratory of Structural Chemistry, Fujian Institute of Research on the Structure of Matter, Chinese Academy of Sciences, Fuzhou 350002, P. R. China
4. Functional Materials and Interfaces Lab, Suzhou Institute of Nano-Tech and Nano-Bionics, Chinese Academy of Sciences, Suzhou 215123, P. R. China
5. School of Nano-Tech and Nano-Bionics, University of Science and Technology of China, Hefei 230026, P. R. China

*These authors contributed equally: Heming Liu, Ruikuan Xie

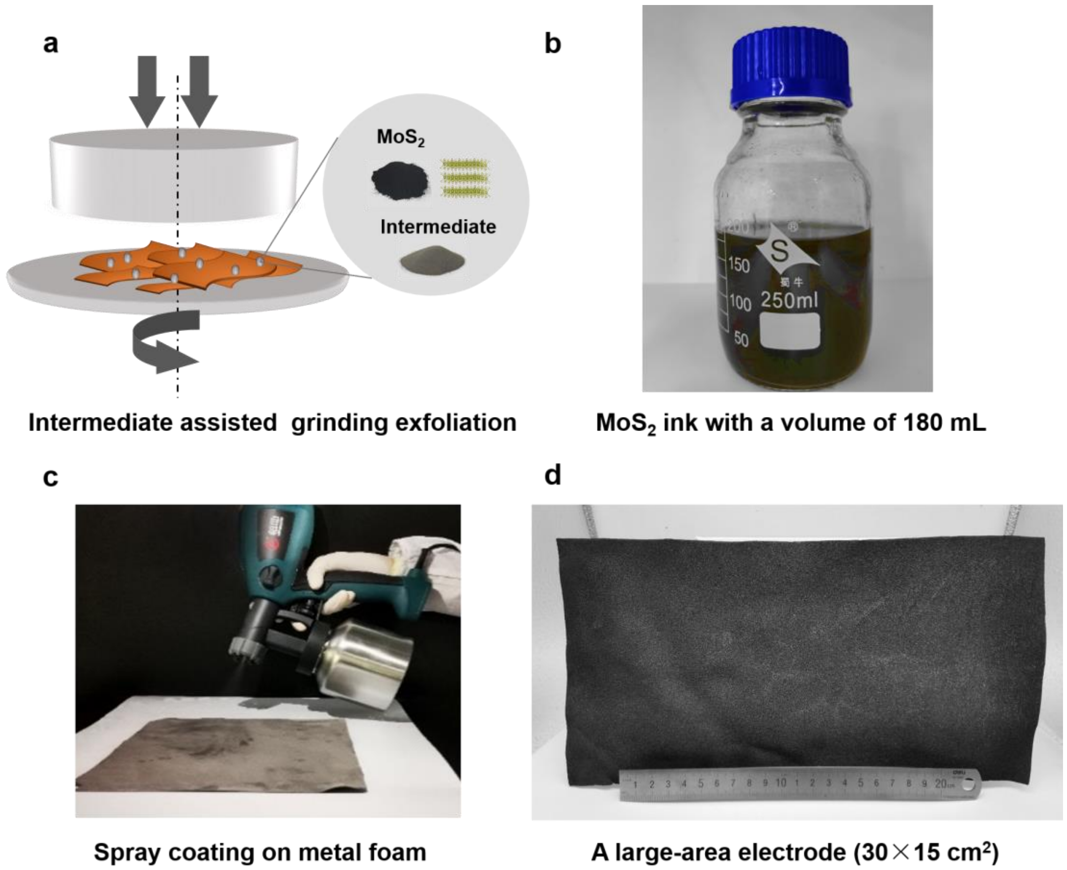

Supplementary Fig. 1 Production of the $CuMo_6S_8$/Cu electrode. a Schematic of the intermediate-assisted grinding method to prepare 2D MoS_2 from bulk MoS_2. **b** A photo of MoS_2 ink dispersed in ethanol. **c** Spray coating of MoS_2 inks onto metal foams. **d** A photo of a $CuMo_6S_8$/Cu electrode with a size of 30 x 15 cm^2.

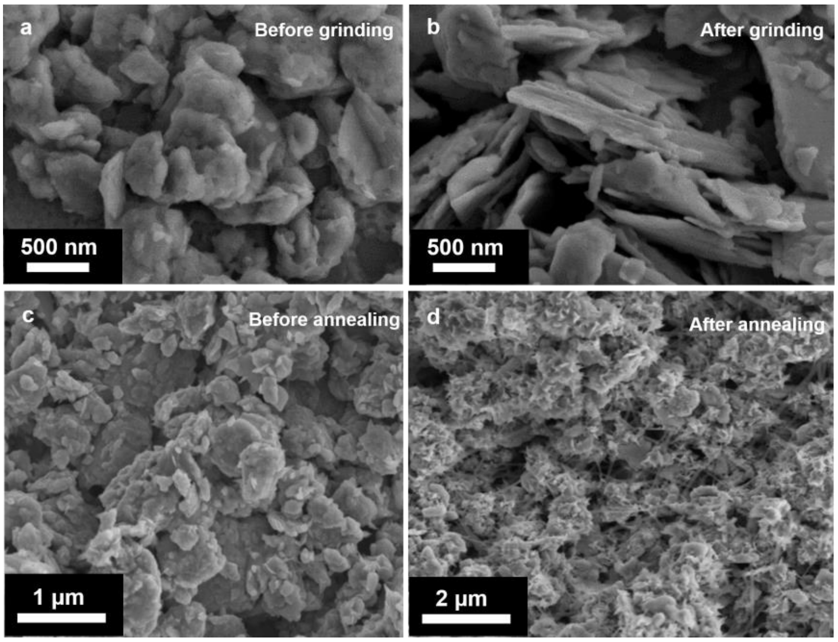

Supplementary Fig. 2 SEM characterization of electrocatalysts. a Bulk MoS_2 before grinding, and (**b**) after grinding. 2D MoS_2 loaded onto Cu foam (**c**) before and (**d**) after annealing.

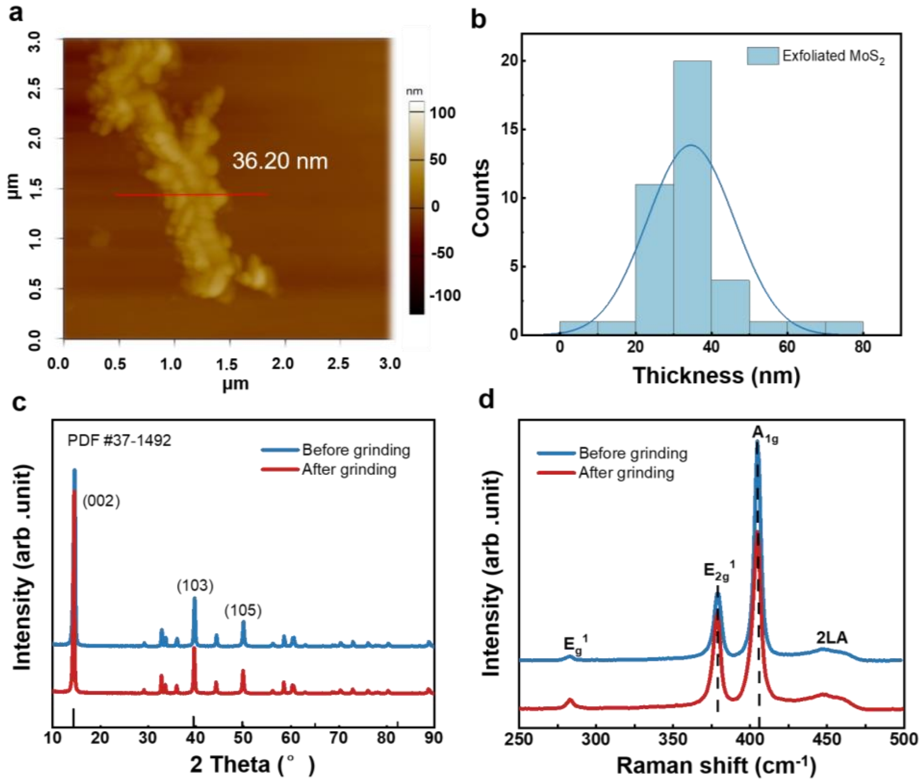

Supplementary Fig. 3 Characterization of the exfoliated 2D MoS_2. a AFM image of 2D MoS_2. **b** statistical data of thickness. **c** XRD patterns and (**d**) Raman spectra of MoS_2 before and after grinding.

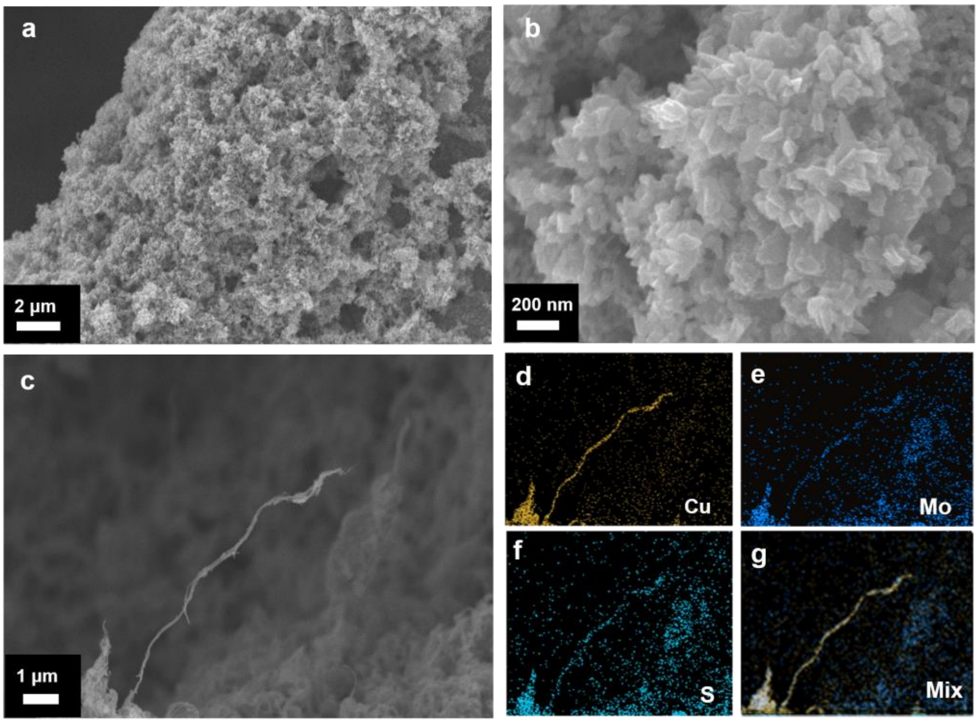

Supplementary Fig. 4 SEM images of the $CuMo_6S_8$/Cu electrode. a, b The porous structure is formed by stacking of nanosheets or nanoparticles, and mixed with nanowires (**c**). **d-g** EDS element mappings of the $CuMo_6S_8$/Cu nanowire.

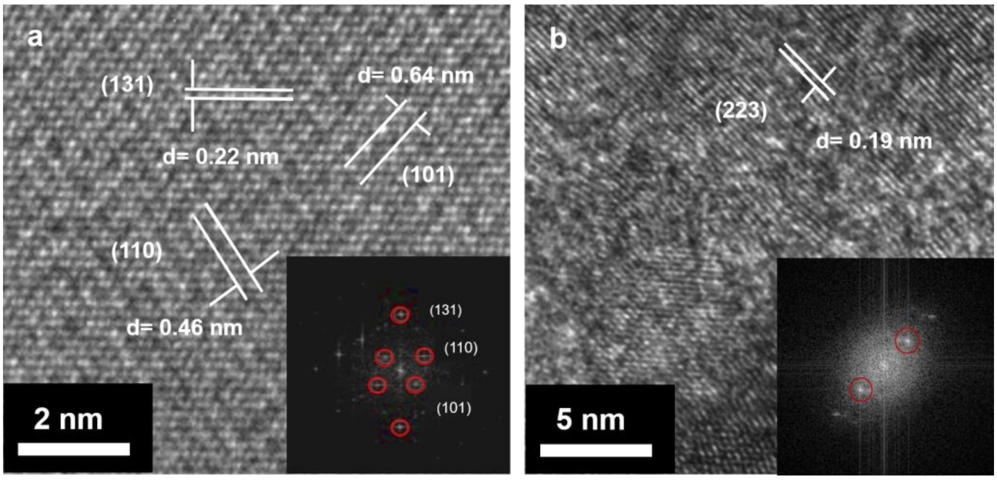

Supplementary Fig. 5 TEM images of the $CuMo_6S_8$/Cu electrode. The lattice planes near the top surface (**a**) and near the interface between $CuMo_6S_8$ and Cu (**b**). The insets are corresponding FFT patterns.

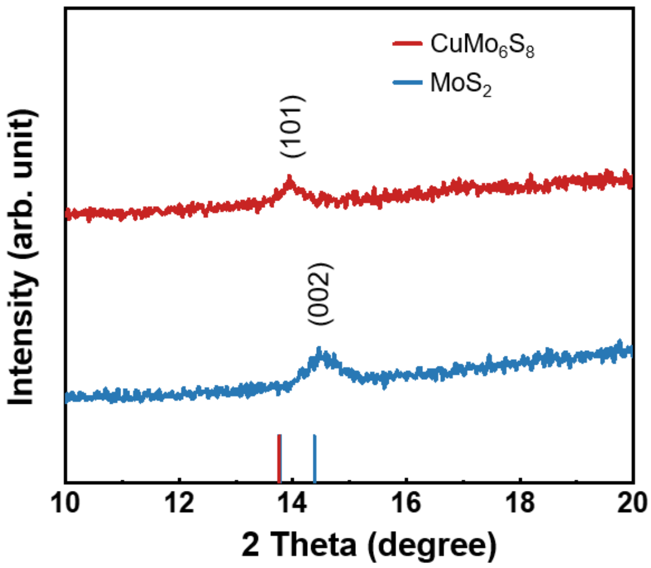

Supplementary Fig. 6 Phase characterization. The zoom-in XRD patterns in the 2 theta range of 10-20 °.

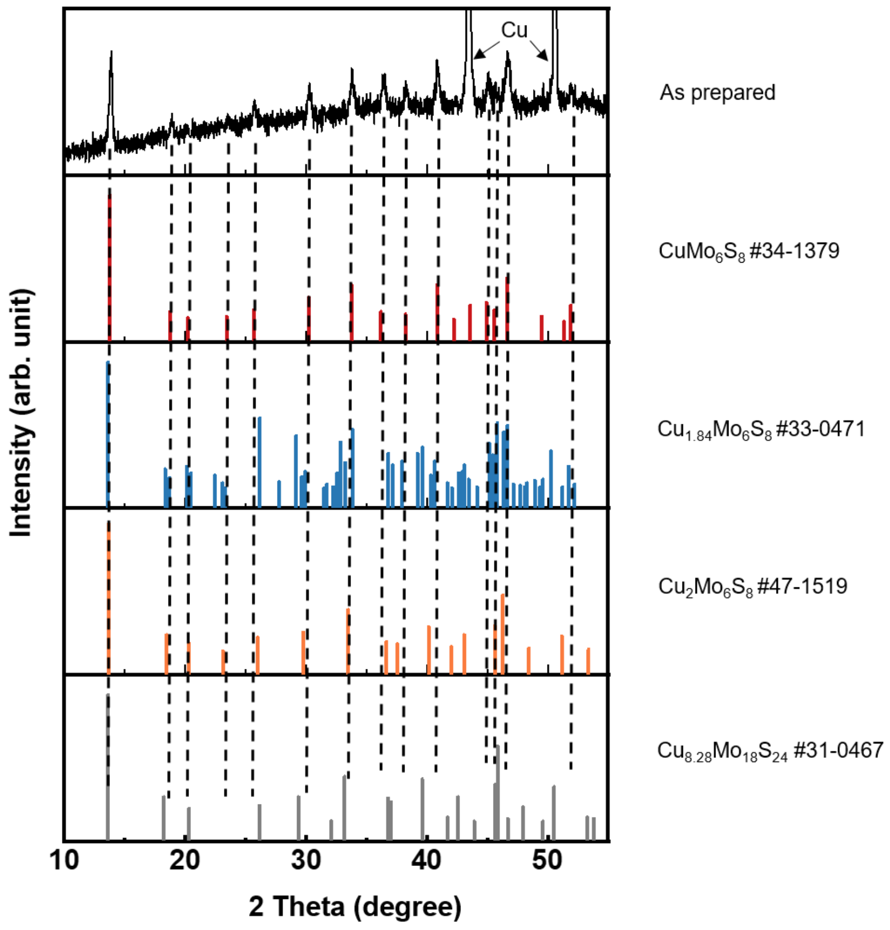

Supplementary Fig. 7 Phase characterization. The comparison of XRD patterns between the as-prepared sample and Chevrel phases, including $CuMo_6S_8$, $Cu_{1.84}Mo_6S_8$, $Cu_2Mo_6S_8$, $Cu_{8.28}Mo_{18}S_{24}$.

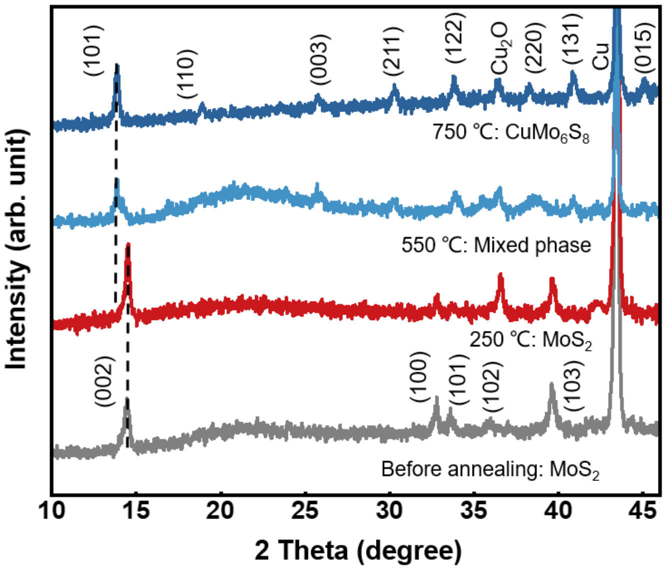

Supplementary Fig. 8 Phase characterization. X-ray diffraction patterns of the samples before annealing, annealed at 250 ℃, 550 ℃, and 750 ℃.

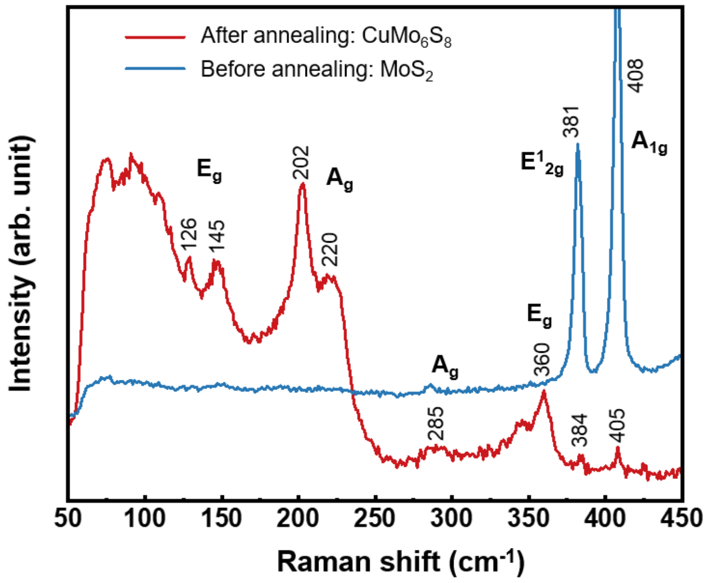

Supplementary Fig. 9 Raman characterization. Raman spectra of the samples before and after annealing.

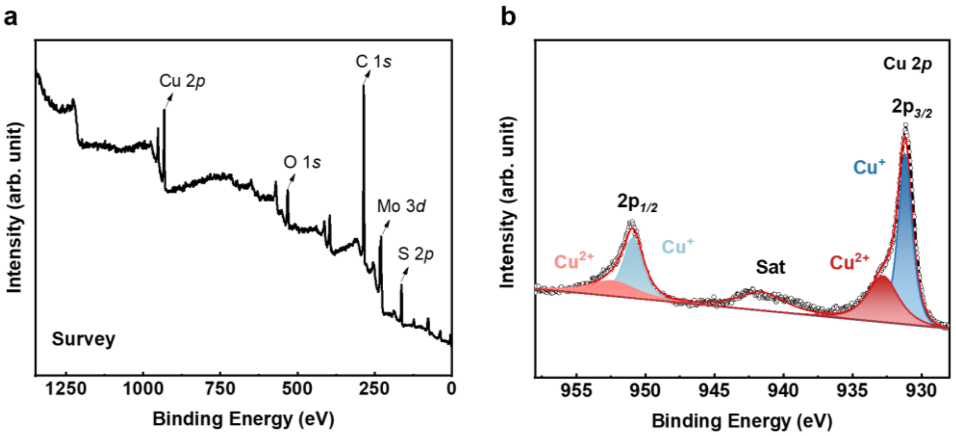

Supplementary Fig. 10 The XPS spectra of the $CuMo_6S_8$/Cu electrode. a Survey spectra, **b** Cu 2*p* spectra.

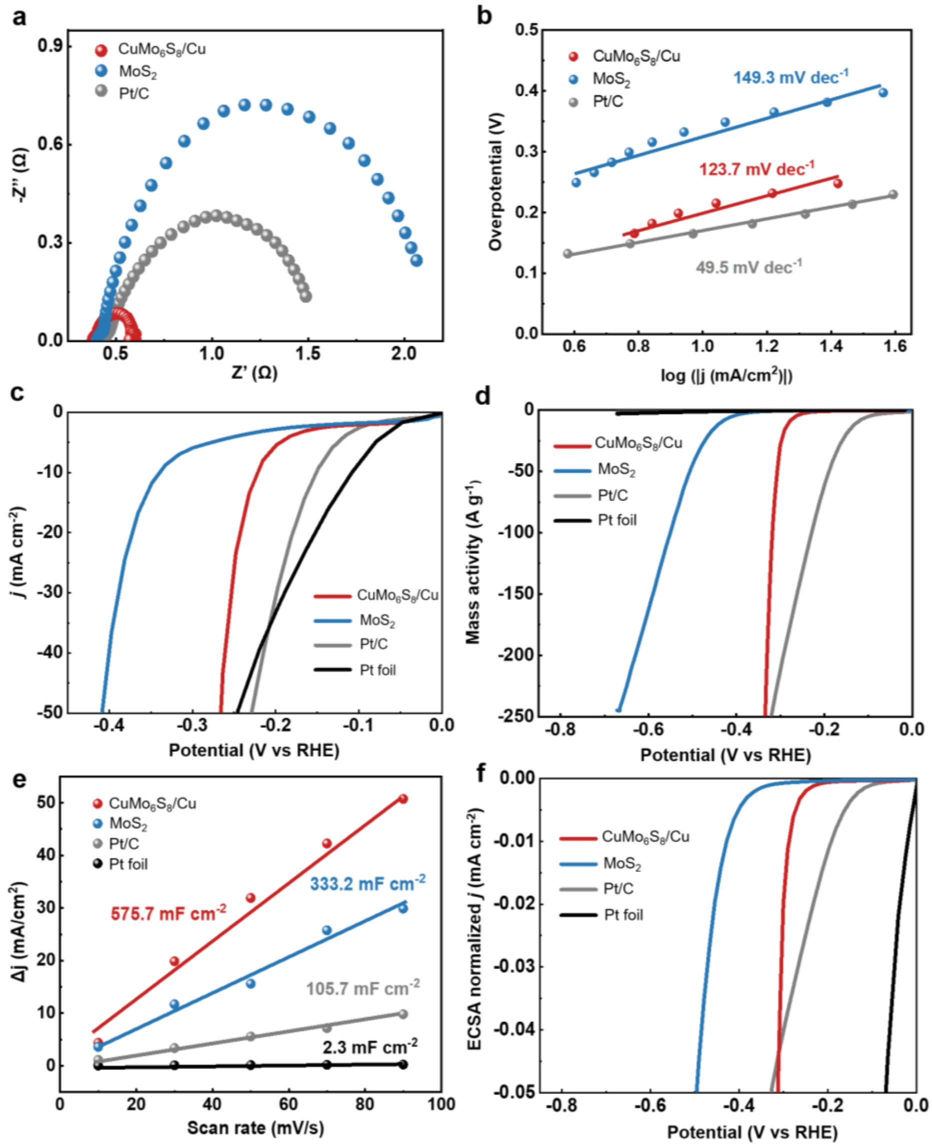

Supplementary Fig. 11 Electrochemical test results of the $CuMo_6S_8$/Cu, MoS_2,

Pt/C and Pt foil electrodes. a EIS, **b** Tafel plots, **c** LSV curves at the range of small current density, **d** Mass activity, **e** Capacitive currents against the scan rate and corresponding C_{dl} values, and **f** ECSA normalized LSV curves.

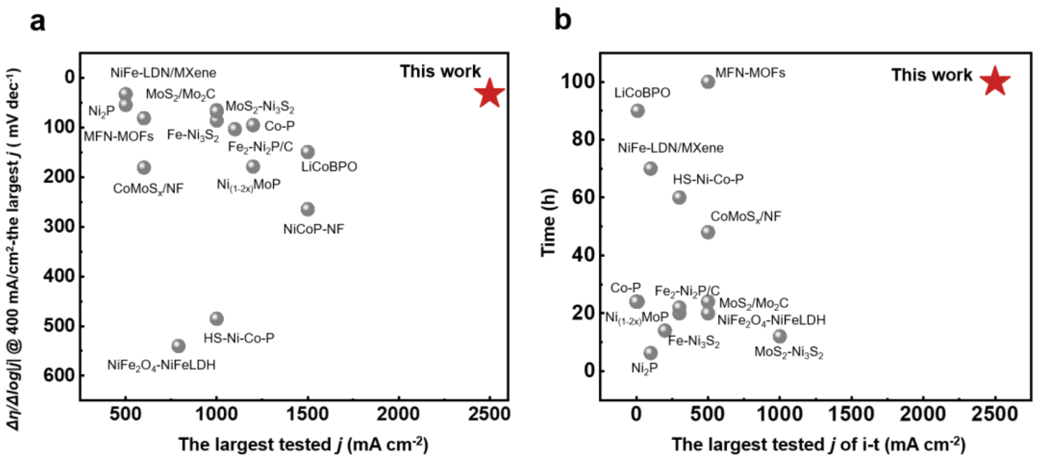

Supplementary Fig. 12 Comparisons of the HER performance and stability of the $CuMo_6S_8$/Cu electrode with non-noble metal electrocatalysts operating at a current density of 500 -1500 mA cm^{-2}. a The reported largest tested current densities and the $\Delta\eta/\Delta\log|j|$ ratio at the range of 400 mA cm^{-2} to the largest tested current density. **b** The largest tested current density of i-t test and their corresponding time.

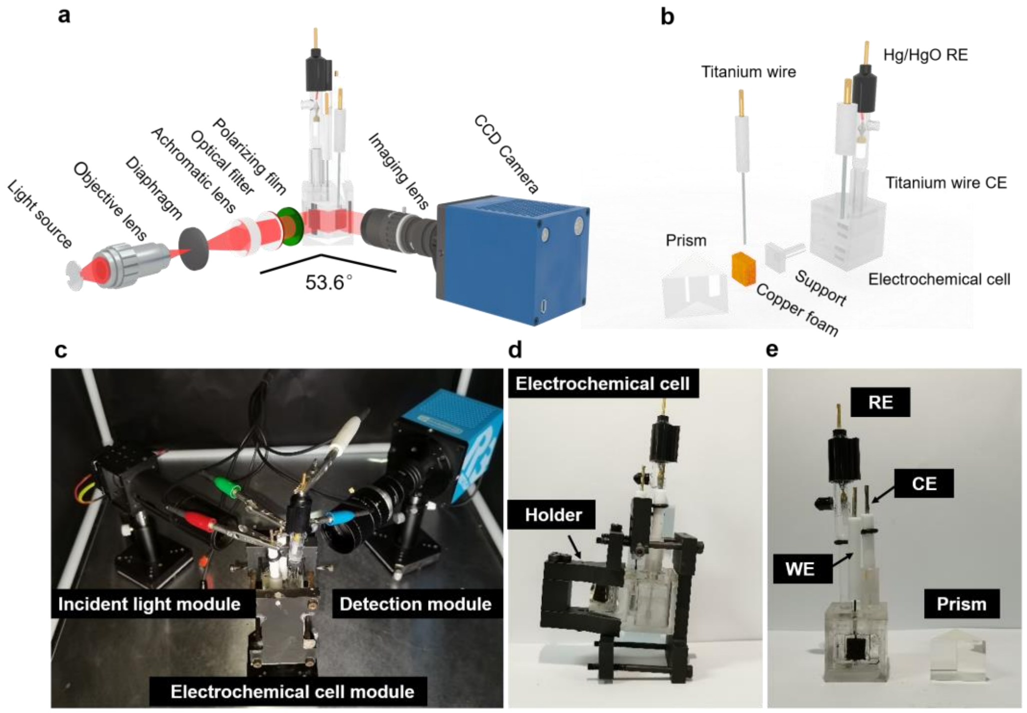

Supplementary Fig. 13 In-situ TIR imaging setup. a The schematic of TIR test system. **b** The explosive view of the electrochemical cell module. **c-e** Photos of the TIR system (**c**), the electrochemical cell module with holder (**d**), and without holder (**e**). The Figs. 13 (a), (b) were adapted from Ref. [41]. Copyright 2022, Wiley.

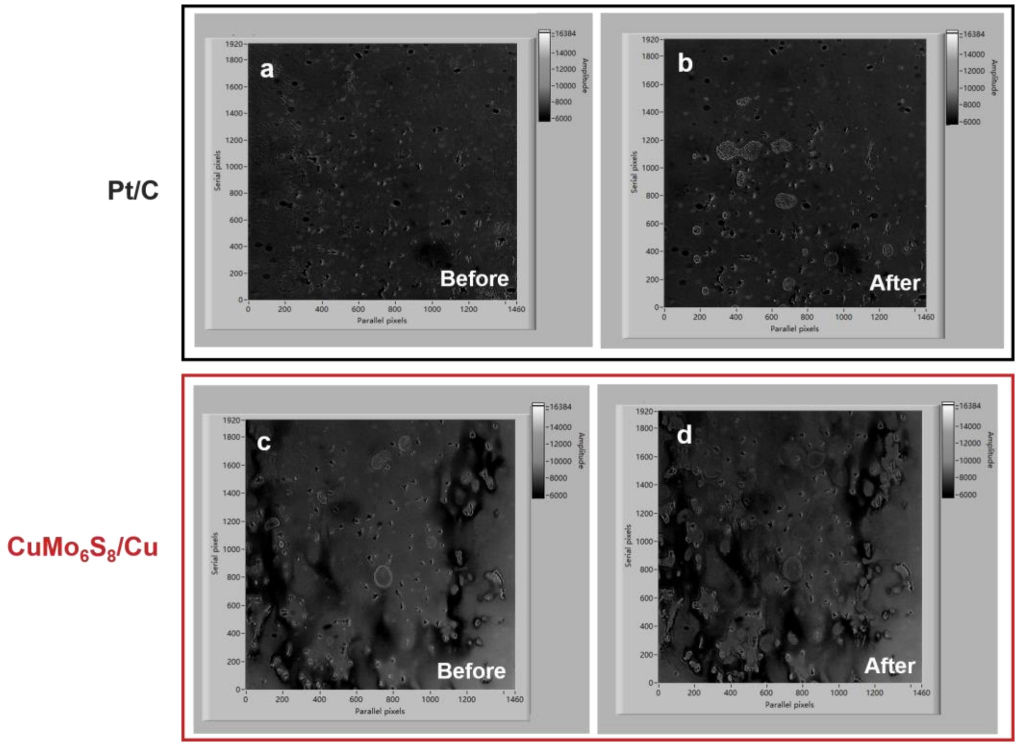

Supplementary Fig. 14. Total internal reflection characterization. The optical

microscopy images of the Pt/C electrode (**a, b**) and $CuMo_6S_8$/Cu electrode (**c, d**) before and after 10,000 CV cycles. The images were taken by the CCD camera of the TIR imaging system.

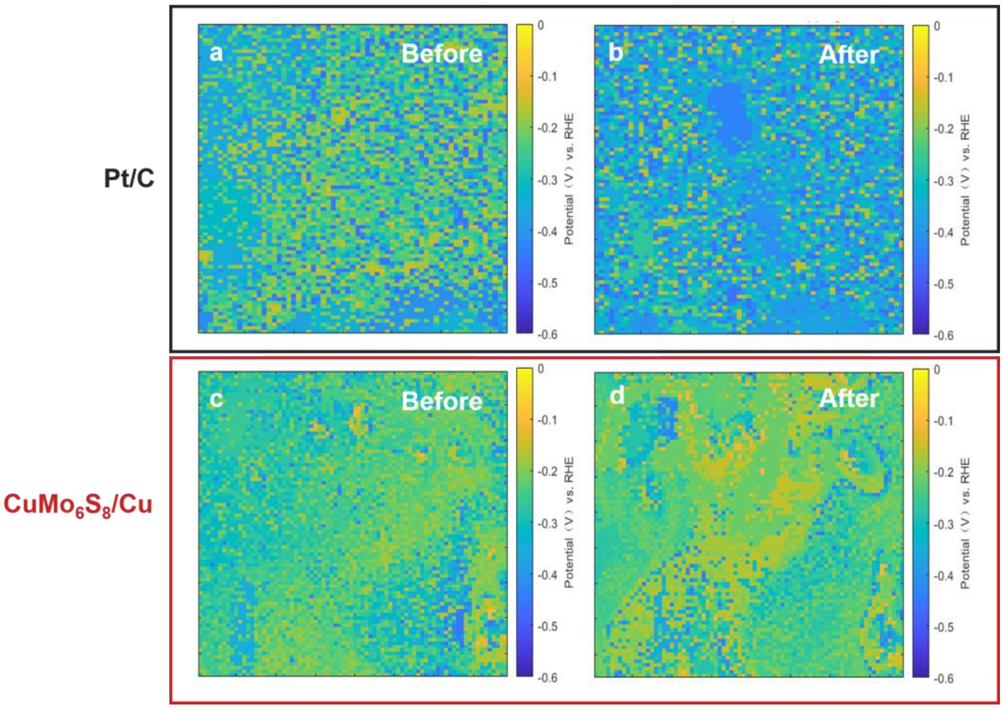

Supplementary Fig. 15 Total internal reflection characterization. The onset potential mappings of the Pt/C (**a, b**) and $CuMo_6S_8$/Cu electrodes (**c, d**) before and after 10,000 CV cycles.

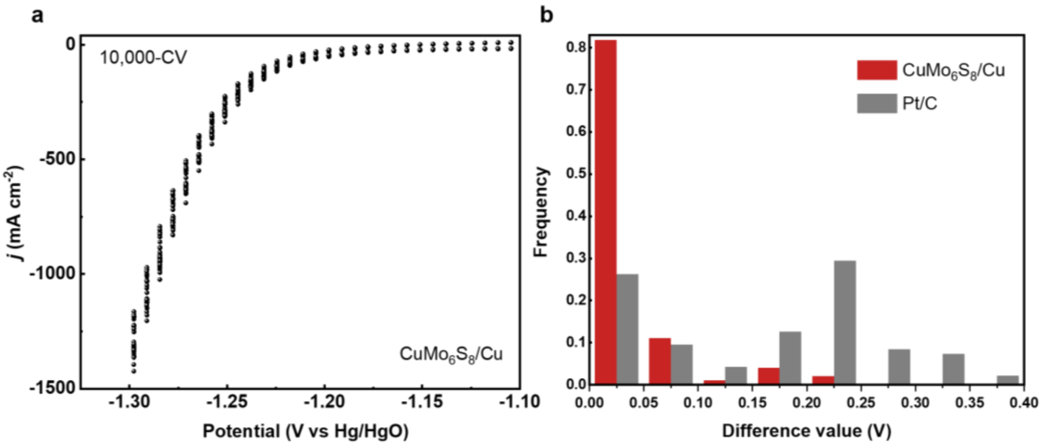

Supplementary Fig. 16 Total internal reflection characterization. TIR method in evaluating performance of electrode with large current density operation. **a** The 10,000

CV cycles of the $CuMo_6S_8$/Cu electrode from 0 to a large current density of -1500 mA cm^{-2}. **b** Statistical data showing the distributions of onset potential differences on $CuMo_6S_8$/Cu and Pt/C electrodes.

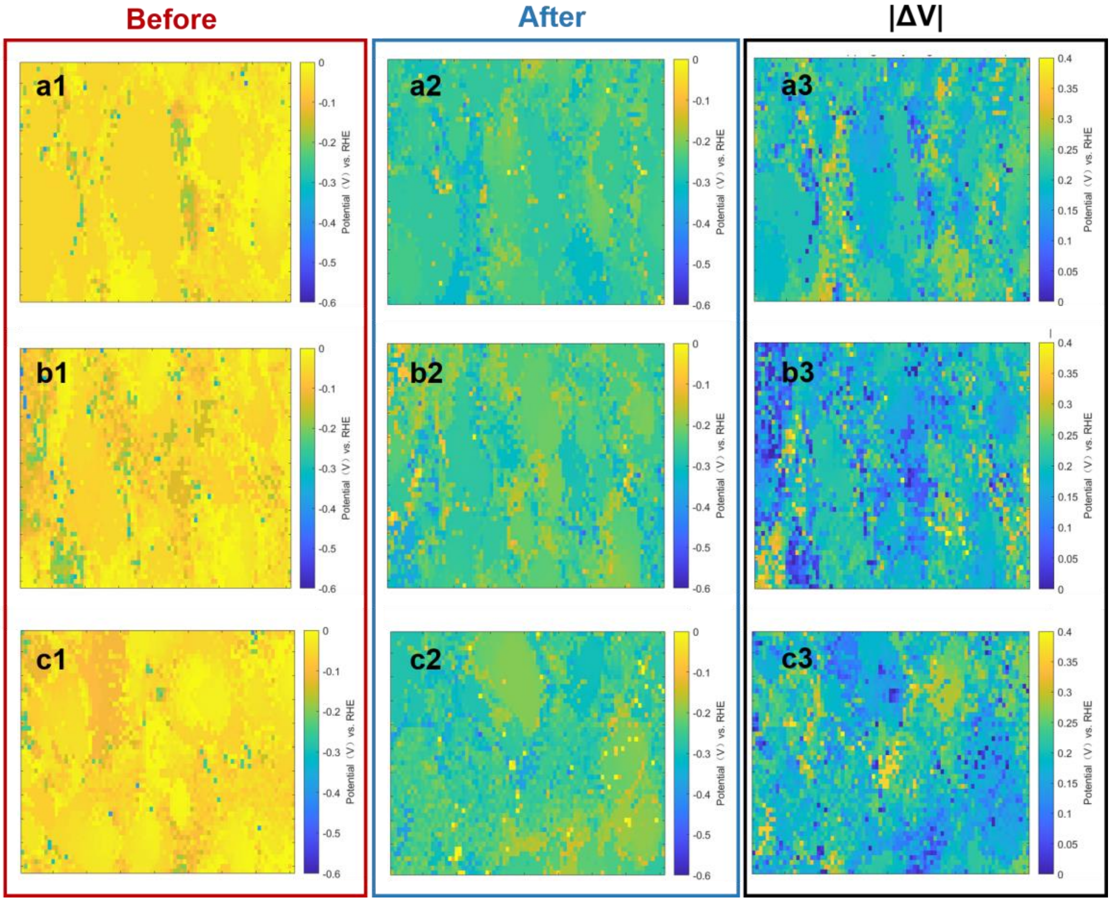

Supplementary Fig. 17 Total internal reflection characterization. The results of three parallel experiments of Pt/C electrodes. The onset potential mapping of three Pt/C electrodes before 10,000 CVs (**a1, b1, c1**), after 10,000 CVs (**a2, b2, c2**) and the absolute differences of on-set potential between the two (**a3, b3, c3**).

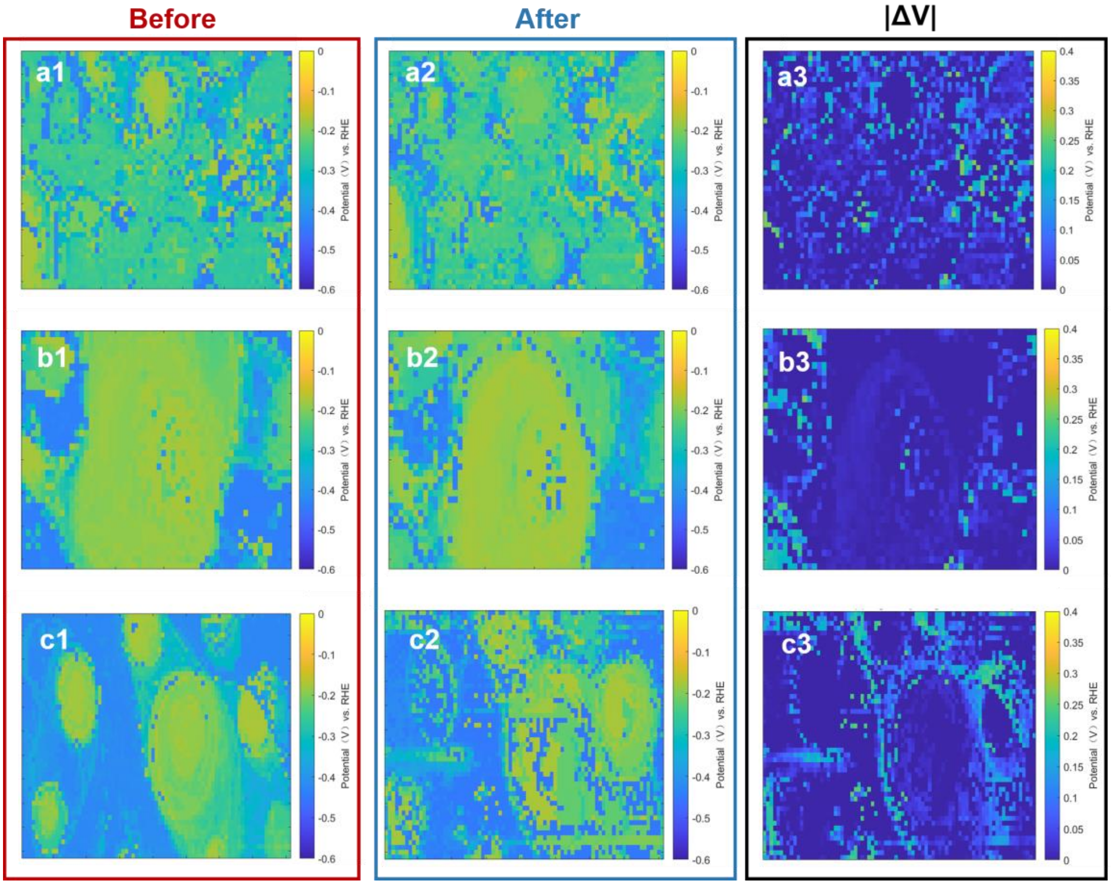

Supplementary Fig. 18 Total internal reflection characterization. The results of three parallel experiments of $CuMo_6S_8$/Cu electrodes. The onset potential mapping of three $CuMo_6S_8$/Cu electrodes before 10,000 CVs (**a1, b1, c1**), after 10,000 CVs (**a2, b2, c2**), and the absolute differences of on-set potential between the two (**a3, b3, c3**).

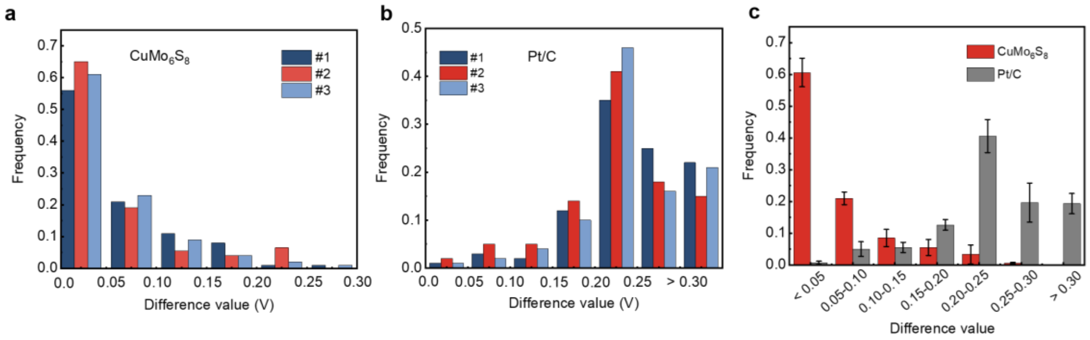

Supplementary Fig. 19 Total internal reflection characterization. Statistics of absolute differences of onset potential from three parallel experiments on $CuMo_6S_8$/Cu (**a**), Pt/C (**b**) electrodes, and their comparison (**c**). The error bars represent the statistical distribution of three samples.

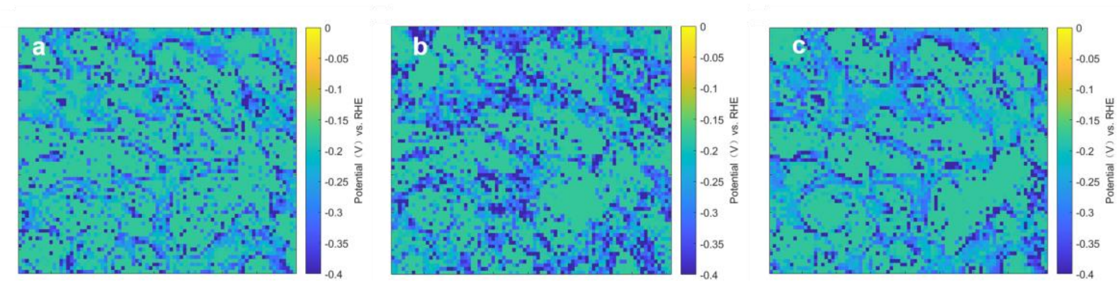

Supplementary Fig. 20 Total internal reflection characterization. The on-set potential mapping results of three replicate measurements (**a**-**c**) for the same $CuMo_6S_8$/Cu electrode.

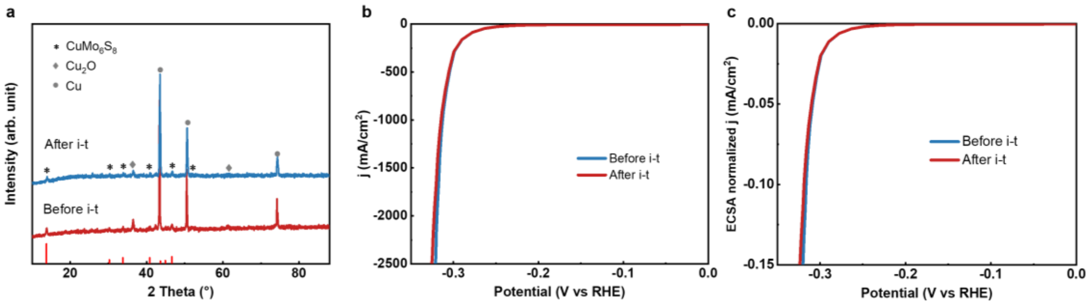

Supplementary Fig. 21 The characterization of the $CuMo_6S_8$/Cu electrode before and after 300 h i-t test. a XRD patterns. **b** LSV curves. **c** ECSA normalized LSV curves.

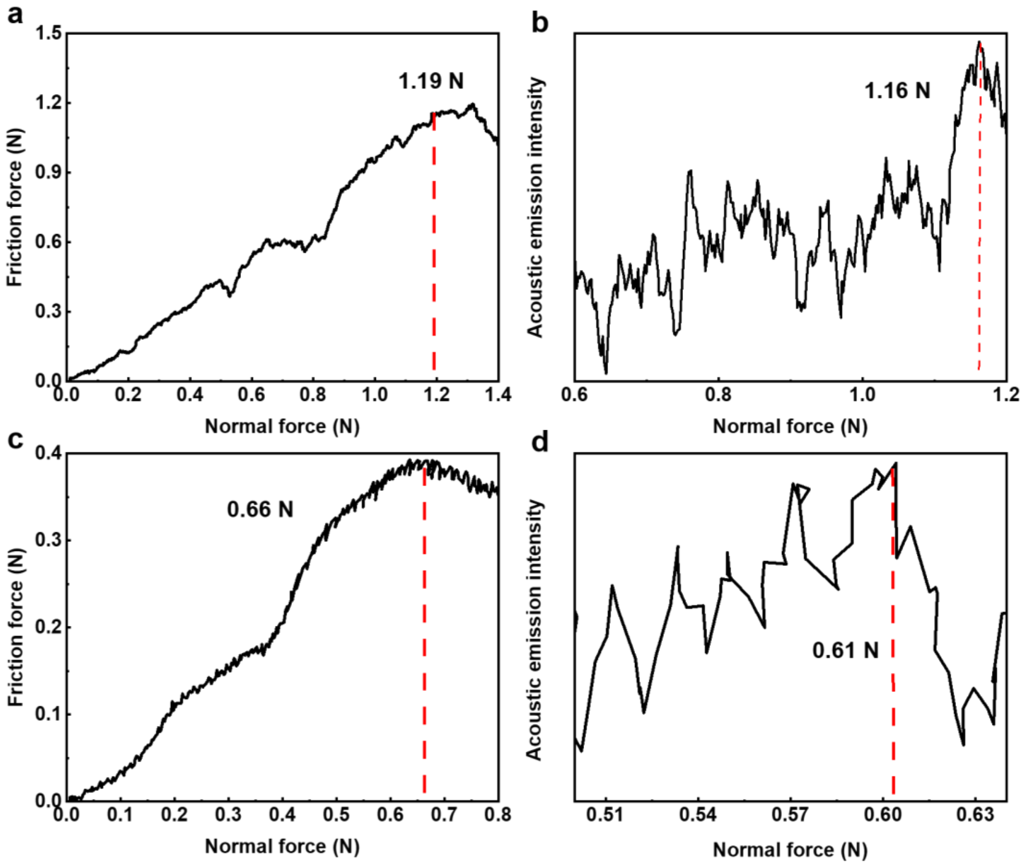

Supplementary Fig. 22 Mechanical characterization. The friction force-normal force curves and acoustic signals of the $CuMo_6S_8$/Cu electrode (**a, b**) and the Pt/C electrode (**c, d**).

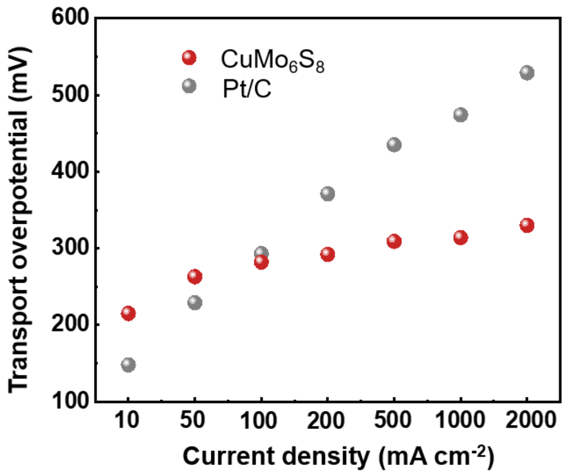

Supplementary Fig. 23 Electrochemical characterization. Transport overpotential of the $CuMo_6S_8$/Cu electrode and Pt/C electrode at different current densities.

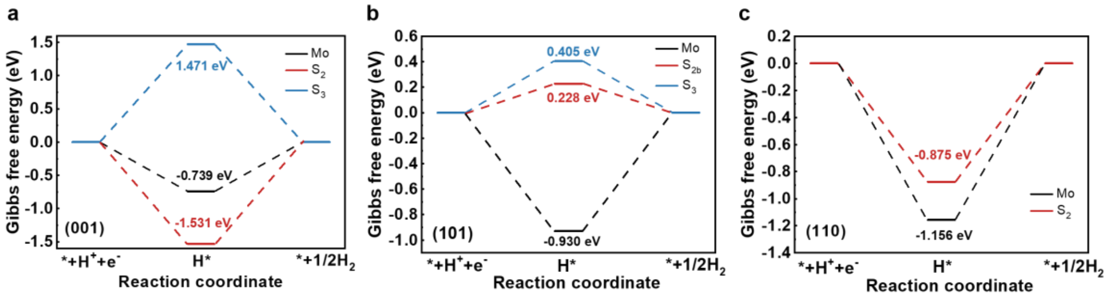

Supplementary Fig. 24 Density functional theory calculation. The ΔG_{H^*} diagrams of active sites in three facets of $CuMo_6S_8$, including (**a**) (001), (**b**) (101), and (**c**) (110).

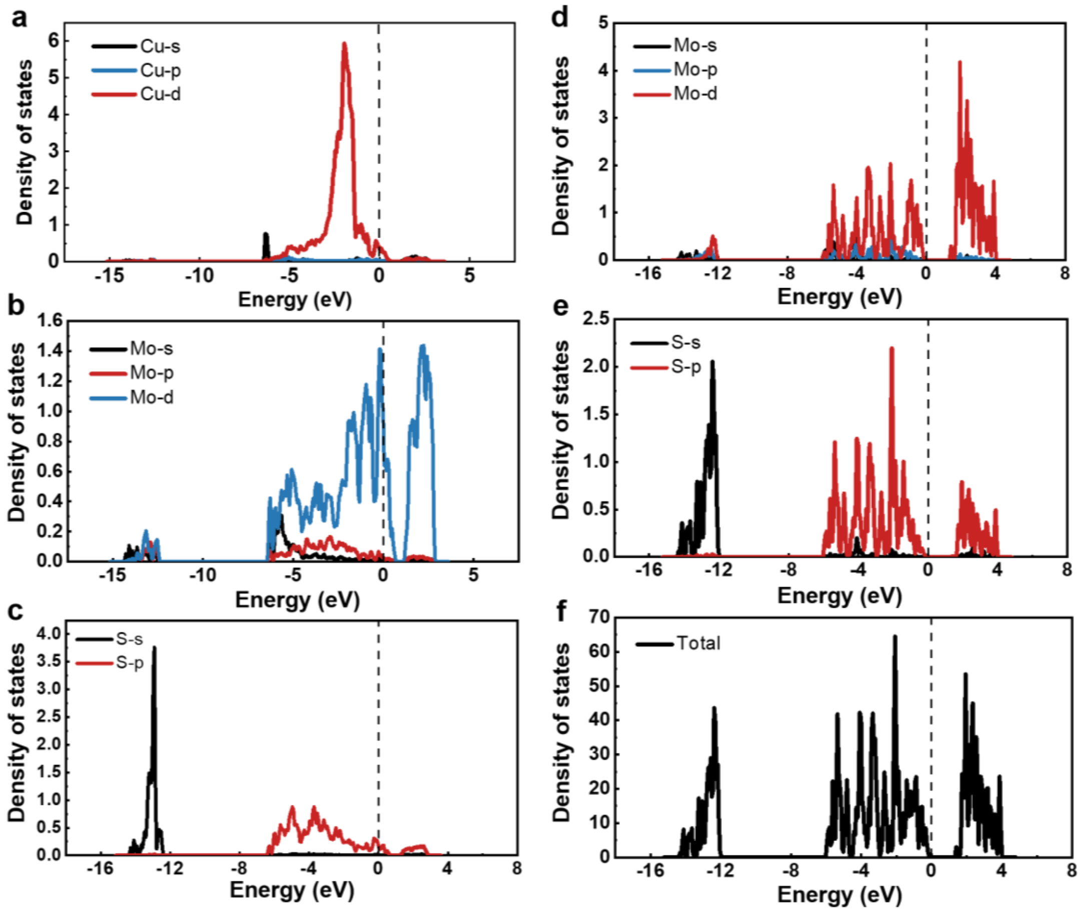

Supplementary Fig. 25 Density functional theory calculation. The PDOS of $CuMo_6S_8$ and MoS_2, including Cu, Mo and S elements of $CuMo_6S_8$ (**a-c**), Mo and S elements of MoS_2 (**d-f**).

Supplementary Table 1. The ICP-OES results of the $CuMo_6S_8/Cu$ electrode.

Sample	Content (moL/kg)		
	**Mo**	**S**	**Mo/S**
$CuMo_6S_8/Cu$	3510.67	4716.34	0.73

Supplementary Table 2. HER performance of Pt/C electrode at large current density.

**Electrocatalysts**	***η* (mV) @ 1000 mA/cm^2**	**References**
Pt/C-NF	490	21
Pt/C-NF	780	22
Pt/C-NF	650 @ 400 mA cm^{-2}	23
Pt/C-NF	370 @ 500 mA cm^{-2}	26
Pt/C-NF	350 @ 460 mA cm^{-2}	28
Pt/C	520 @ 600 mA cm^{-2}	29
Pt/C-NF	420	33
Pt/C	240 @ 400 mA cm^{-2}	36
Pt/C	230 @ 800 mA cm^{-2}	37
Pt/C	410	9
**Pt/C-Cu foam**	**450**	**This work**

Supplementary Table 3. Comparisons of HER performance and stability of the Cu/$CuMo_6S_8$ electrode with non-noble metal electrocatalysts operating at above 2000 mA cm^{-2}.

**Catalysts**	**Δη/Δlog|j| @ 1000 mA cm^{-2} - 2000 mA cm^{-2}**	**η (mV) @ 2000 mA/cm^2**	**The largest tested j of i-t test (mA/cm^2)**	**Time (h)**	**Refs**
$Nb_{1.35}S_2$	212.9	360	-	-	20
Ni_2P/NF	403.1	417	2500	6	21
α-MoB_2	269.6	413	2000	0.27	22
Nano-KFO/NF	132.4	340	1500	60	23
F-Co_2P/Fe_2P/IF	105.5	300	2000	10	24
Ta/TaS_2	58	400	1000	10	25
**$CuMo_6S_8$**	**32.5**	**320**	**2500**	**100**	**This work**

Supplementary Table 4. Comparisons of HER performance and stability of the Cu/$CuMo_6S_8$ electrode with other electrocatalysts operating at large current density of 500 - 1500 mA cm^{-2}.

Catalysts	The largest tested j (mA/cm^2)	$\Delta\eta/\Delta\log\|j\|$ @ 400 mA/cm^2- the largest current density	The largest tested j of i-t test (mA/cm^2)	Time (h)	Refs
NiFe-LDN/MXene	500	33	100	70	26
Ni_2P	500	54.7	100	6.25	27
MFN-MOFs	600	81.4	500	100	28
$CoMoS_x$/NF	600	180.8	500	48	29
$NiFe_2O_4$-NiFe LDH	790	540.3	500	20	30
HS-Ni-Co-P	1000	485.3	300	60	31
Fe-Ni_3S_2	1000	65.2	200	14	32
MoS_2-Ni_3S_2	1000	67.6	1000	12	33
MoS_2/Mo_2C	1000	86.2	500	24	9
Fe_2-Ni_2P/C	1100	103.4	300	22	34
$Ni_{(1-2x)}MoP$	1200	178.8	300	20	35
Co-P	1200	95.3	0.5	24	36
NiCoP-NF	1500	264.6	10	24	37
LiCoBPO	1500	149.6	10	90	38
**$CuMo_6S_8$**	**2500**	**32.5**	**2500**	**100**	**This work**

Supplementary Table 5. The ICP-OES results of the dissolved metals from the $CuMo_6S_8$/Cu electrode in electrolyte after i-t test.

Sample	Content (moL/L)		
	**Cu**	**Mo**	**S**
Solution (After i-t test)	< 0.0001	0.0003	0.022

Supplementary Table 6. Parameters for ECSA normalized LSV curves of the $CuMo_6S_8$ electrode before and after i-t test.

	η@-0.0005 mA cm^{-2} (ECSA)	η@-0.001 mA cm^{-2} (ECSA)	η@-0.01 mA cm^{-2} (ECSA)	η@-0.1 mA cm^{-2} (ECSA)
Before i-t	-0.1842	-0.2962	-0.2897	-0.3166
After i-t	-0.1843	-0.2971	-0.29	-0.3195
Δ(%)	0.05%	0.30%	0.10%	0.91%

Supplementary Table 7. Volume concentration of H_2 bubble calculated from reflective index (RI) and density resolution.

	1M KOH	H_2	Resolution	Equivalent RI/ Density	**Volume concentration**
RI	1.409	1.0001	8.13E-07[37]	1.4089	**1.99E-06**
Density (g cm^{-3})	1.05	8.90E-05	3.0E-04[38]	1.047	**2.86E-04**

Supplementary Table 8. The Bader effective charge and ΔG_{H*} of sulfur active sites with different coordinations in three facets.

**Facet**	**(001)**		**(101)**			**(110)**	
**S coordinate number**	3	4	2a	2b	3	2	3
**Bader effect charge |e|**	-0.65	-0.74	0.49	-0.72	-0.75	0.55	-0.66
**ΔG_{H*} (eV)**	-0.08	0.45	0.22	0.21	0.40	0.87	0.20

Supplementary Note 1

When there are gas bubbles generated on the catalysts, whether the bubbles can adhere to the catalyst or not, and the firmness of the adhesion depends on the change of the interfacial energy before and after adhesion. If the total interfacial energy is reduced, it can be attached and vice versa. The more the energy is reduced, the stronger the adhesion is. Before the gas bubbles attached to electrocatalysts, the interfacial energy per unit area W_1 is equal to the sum of gas-liquid interfacial energy σ_{GL} and liquid-solid interfacial energy σ_{LS}, that $W_1 = \sigma_{GL} + \sigma_{LS}$. After attachment, since there is no solid-liquid interface in the attachment region, the total interfacial energy per unit area is changed to W_2, that $W_2 = \sigma_{GS}$. Therefore, the difference of total interfacial energy before and after attachment is that $\Delta W = W_2 - W_1 = \sigma_{GS} - \sigma_{GL} - \sigma_{LS}$.

According to the Young's equation that $\sigma_{GS} = \sigma_{LS} + \sigma_{GL} cos\theta$, the result of ΔW can be written as $\Delta W = \sigma_{GL}(cos\theta - 1)$. Therefore, when the electrocatalysts are superhydrophilic, that $\theta = 0°$, $\Delta W = 0$, the gas bubbles can not be attached to it. When the electrocatalysts are superhydrophobic, that $\theta = 180°$, $\Delta W = -2\sigma_{GL}$, the gas bubbles tend to be attached to it.

Supplementary Note 2

The total overpotential η_{total} consists of activation overpotential of HER η_{act}, cell ohmic overpoetntial $\eta_{ohm, cell}$ and transport overpotential caused by gas bubble η_{tra}. The η_{tra} mainly includes three parts: the overpotential of ohmic loss $\eta_{ohm, bubble}$ caused by a blockage of the ion pathways, the concentration overpotential η_{con} caused by bubble-induced convection and variation of supersaturation in electrolyte and the activation overpotential $\eta_{act, bubble}$ caused by coverage of active sites. These relationships are summarized in Equation (3):

$$\eta_{\text{total}} = \eta_{\text{act}} + \eta_{\text{ohm,cell}} + \eta_{\text{tra}} \quad (3)$$

$$= \eta_{\text{act}} + \eta_{\text{ohm,cell}} + \eta_{\text{ohm,bubble}} + \eta_{\text{con}} + \eta_{\text{act,bubble}}$$

Therefore, the η_{tra} can be achieved by subtracting the η_{act} and $\eta_{ohm, cell}$. They can be approximately determined by onset potential and EIS, respectively.

Supplementary Note 3

The detection sensitivity for hydrogen bubble formation could be characterized by volume concentration of H_2 bubbles. The relationship between equivalent RI/density and volume concentration of H_2 bubbles follow the equations below[41]:

$$n_{\text{equivalent}} = (1 - v) \times n_{\text{electrolyte}} + v \times n_{\text{bubble}} \quad (1)$$

$$\rho_{\text{equivalent}} = (1 - v) \times \rho_{\text{electrolyte}} + v \times \rho_{\text{bubble}} \quad (2)$$

Where n is the RI, ρ is the density and the v is the volume concentration of H_2 bubbles. Amongst, the RI and density of 1M KOH and H_2 are known, and the RI resolution and density resolution of TIR and X-ray imaging methods are obtained from literature, respectively. Therefore, the equivalent RI and equivalent density can be obtained by subtracting the RI resolution and density resolution from the RI and density of KOH. Finally, the volume concentration of H_2 bubbles could be obtained according to the equation (1) and (2). The density resolution of X-ray imaging is in the range of 0.3-10 mg cm^{-3}. Taking the density resolution of 0.3 mg cm^{-3} as an example, the detectable H_2 volume concentration only reaches to 2.86×10^{-4} (Supplementary Table 5), which means the H_2 bubble detection sensitivity of X-ray imaging is two orders of

magnitude lower than that of TIR (1.99 $\times 10^{-6}$). Therefore, it is more accurate for our method to obtain onset potential of electrode than X-ray imaging.

Supplementary References

1. Deng, H., Zhang, C., Xie, Y. C., et al. Laser induced MoS_2/carbon hybrids for hydrogen evolution reaction catalysts. *J. Mater. Chem. A* **4**, 6824-6823 (2016).
2. Jian, J., Li, Y., Bi, H., et al. Aluminum decoration on MoS_2 ultrathin nanosheets for highly efficient hydrogen evolution. *ACS Sustainable Chem. Eng.* **8**, 4547-4554 (2020).
3. Anjum R. M. A., Jeong H. Y., Lee, M. H., et al. Efficient hydrogen evolution reaction catalysis in alkaline media by all-in-one MoS_2 with multifunctional active sites. *Adv. Mater.* **30**, 1707105 (2018).
4. Wang, S., Zhang, D., Li, B., et al. Ultrastable in-plane 1T-2H MoS_2 heterostructures for enhanced hydrogen evolution reaction. *Adv. Energy Mater.* **8**, 1801345 (2018).
5. Bai, L., Gao, L., Conway, B. E. Problem of in situ real-area determination in evaluation of performance of rough or porous, gas-evolving electrocatalysts. Part 1. Basis for distinction between capacitance of the double layer and the pseudocapacitance due to adsorbed H in the H_2 evolution reaction at Pt. *J. Chem. Soc., Faraday Trans.* **89**, 235-242 (1993).
6. Fournier, J., Brossard, L., Tilquin, J.Y., et al. Hydrogen evolution reaction in alkaline solution: catalytic influence of Pt supported on graphite *vs.* Pt inclusions in graphite. *J. Electrochem. Soc.* **143**, 919-926 (1996).
7. Glarum, S. H., Marshall, J. H. An A-C admittance study of the platinum/sulfuric acid interface. *J. Electrochem. Soc.* **126**, 424-430 (1979).
8. McCrory, C. C. L., Jung, S., Peters, J. C., et al. Benchmarking heterogeneous electrocatalysts for the oxygen evolution reaction. *J. Am. Chem. Soc.* **135**, 16977-16987 (2013).
9. Zhang, C. et al. High-throughput production of cheap mineral-based two-dimensional electrocatalysts for high-current-density hydrogen evolution. *Nat. Commun.* **11**, 1-8 (2020).

10. Hohenberg, P., Kohn, W. Inhomogeneous electron gas. *Phys. Rev.* **136**, B864-B871 (1964).
11. Kresse, G., Furthmüller, J. Efficient iterative schemes for ab initio total-energy calculations using a plane-wave basis set. *Phys. Rev. B* **54**, 11169-11186 (1996).
12. Kresse, G., Furthmüller, J. Efficiency of ab-initio total energy calculations for metals and semiconductors using a plane-wave basis set. *Comput. Mater. Sci.* **6**, 15-50 (1996).
13. Kresse, G., Hafner, J. Ab initio molecular-dynamics simulation of the liquid-metal--amorphous-semiconductor transition in germanium. *Phys. Rev. B* **149**, 14251-14269 (1994).
14. Blöchl, P. E. Projector augmented-wave method. *Phys. Rev. B* **50**, 17953-17979 (1994).
15. Perdew, J. P., Burke, K., Ernzerhof, M. Generalized gradient approximation made simple. *Phys. Rev. Lett.* **77**, 3865-3868 (1996).
16. Monkhorst, H. J., Pack, J. D. Special points for Brillouin-zone integrations. *Phys. Rev. B* **13**, 5188-5192 (1976).
17. Nørskov, J. K., Rossmeisl, J., Logadottir, A., Lindqvist, L., Kitchin, J. R., Bligaard, T., Jonsson, H. Origin of the overpotential for oxygen reduction at a fuel-cell cathode. *J. Phys. Chem. B* **108**, 17886-17892 (2004).
18. Chai, G.-L., Hou, Z., Shu, D.-J., Ikeda, T., Terakura, K. Active sites and mechanisms for oxygen reduction reaction on nitrogen-doped carbon alloy catalysts: Stone-Wales defect and curvature effect. *J. Am. Chem. Soc.* **136**, 13629-13640 (2014).
19. Chai, G.-L., Qiu, K., Qiao, M., Titirici, M.-M., Shang, C., Guo, Z. Active sites engineering leads to exceptional ORR and OER bifunctionality in P, N Co-doped graphene frameworks. *Energy Environ. Sci.* **10**, 1186-1195 (2017).
20. Yang, J. et al. Ultrahigh-current-density niobium disulfide catalysts for hydrogen evolution. *Nat. Mater.* **18**, 1309-1314 (2019).
21. Yu, X. et al. "Superaerophobic" nickel phosphide nanoarray catalyst for efficient hydrogen evolution at ultrahigh current densities. *J. Am. Chem. Soc.* **141**, 7537-

7543 (2019).

22. Chen, Y. et al. Highly active, nonprecious electrocatalyst comprising borophene subunits for the hydrogen evolution reaction. *J. Am. Chem. Soc.* **139**, 12370-12373 (2017).
23. Jian, J. et al. Metal-ionic-conductor potassium ferrite nanocrystals with intrinsic superhydrophilic surfaces for electrocatalytic water splitting at ultrahigh current densities. *J. Mater. Chem. A* **9**, 7586-7593 (2021).
24. Zhang, X. et al. Hydrogen evolution under large-current-density based on fluorine-doped cobalt-iron phosphides. *Chem. Eng. J.* **399**, 125831 (2020).
25. Yu, Q. et al. A Ta-TaS_2 monolith catalyst with robust and metallic interface for superior hydrogen evolution. *Nat. Commun.* **12**, 1-8 (2021).
26. Yu, M., Wang, Z., Liu, J., Sun, F., Yang, P., Qiu, J. A hierarchically porous and hydrophilic 3D nickel-iron/MXene electrode for accelerating oxygen and hydrogen evolution at high current densities. *Nano Energy* **63**, 103880 (2019).
27. Cai, L. et al. Active site engineering of Fe-and Ni-sites for highly efficient electrochemical overall water splitting. *J. Mater. Chem. A* **6**, 21445-21451 (2018).
28. Raja, D. S., Lin, H. W., Lu, S. Y. Synergistically well-mixed MOFs grown on nickel foam as highly efficient durable bifunctional electrocatalysts for overall water splitting at high current densities. *Nano Energy* **57**, 1-13 (2019).
29. Shan, X. et al. An engineered superhydrophilic/superaerophobic electrocatalyst composed of the supported $CoMoS_x$ chalcogel for overall water splitting. *Angew. Chem. Int. Ed.* **59**, 1659-1665 (2020).
30. Wu, Z., Zou, Z., Huang, J., Gao, F. $NiFe_2O_4$ nanoparticles/NiFe layered double-hydroxide nanosheet heterostructure array for efficient overall water splitting at large current densities. *ACS Appl. Mater. Interfaces* **10**, 26283-26292 (2018).
31. Zhang, P. et al. Gas-templating of hierarchically structured Ni-Co-P for efficient electrocatalytic hydrogen evolution. *J. Mater. Chem. A* **5**, 7564-7570 (2017).
32. Wang, X., Zhang, W., Zhang, J., Wu, Z. Fe-Doped Ni_3S_2 nanowires with surface-restricted oxidation toward high-current-density overall water splitting. *ChemElectroChem* **6**, 4550-4559 (2019).

33. Xue, S., Liu, Z., Ma, C., Cheng, H. M., Ren, W. A highly active and durable electrocatalyst for large current density hydrogen evolution reaction. *Sci. Bull.* **65**, 123-130 (2020).
34. Sun, H. et al. Morphological and electronic tuning of Ni_2P through iron doping toward highly efficient water splitting. *ACS Catal.* **9**, 8882-8892 (2019).
35. Yu, L. et al. Ternary $Ni_{2(1-x)}Mo_{2x}P$ nanowire arrays toward efficient and stable hydrogen evolution electrocatalysis under large-current-density. *Nano Energy* **53**, 492-500 (2018).
36. Jiang, N., You, B., Sheng, M., Sun, Y. Electrodeposited cobalt-phosphorous-derived films as competent bifunctional catalysts for overall water splitting. *Angew. Chem. Int. Ed.* **127**, 6349-6352 (2015).
37. Yu, C. et al. Bimetallic Ni-Co phosphide nanosheets self-supported on nickel foam as high-performance electrocatalyst for hydrogen evolution reaction. *Electrochim. Acta* **317**, 191-198 (2019).
38. Menezes, P. W. et al. Helical cobalt borophosphates to master durable overall water-splitting. *Energy Environ. Sci.* **12**, 988-999 (2019).
39. K. Ma et al., An optimized angular total internal reflection sensor with high resolution in vanadium flow batteries. *IEEE Trans. Instrum. Meas.* **69**, 3170-3178 (2020).
40. Akio Yoneyama et al. Phase-contrast X-ray imaging system with sub-mg/cm3 density resolution. *J. Phys.: Conf. Ser.* **425**, 192007 (2013).
41. Li, F., Ma, K., Liu, L., Xi, J., Qiu, X. Characterizing the onset potential distribution of Pt/C catalyst deposition by a total internal reflection imaging method. *Small* **17**, 2102407 (2021).
