# Supplementary material for: Dual interfacial engineering of a Chevrel phase electrode material for stable hydrogen evolution at 2500 mA cm−2
Source: Nat Commun. 2022 Oct 26;13:6382. doi: 10.1038/s41467-022-34121-y (PMC9605970; doi:10.1038/s41467-022-34121-y)
Supplement: Supplementary file 3 — Description of Additional Supplementary Files [file 41467_2022_34121_MOESM3_ESM.pdf]

Description of Additional Supplementary Files

File Name: Supplementary Movie 1
Description: A movie showing the intensity of reflection light from the Pt/C electrode surface as a function of applied potential. The left side shows the mappings of light intensity at different potentials. The right side shows the curves of the light intensity at point A and B as a function of potential.

File Name: Supplementary Movie 2
Description: A movie showing the bubble evolution of Pt/C (left) and $CuMo_6S_8$ electrodes (right) at 10 mA cm^{-2}. The scale bar is 0.25 mm.
